# Disordered actomyosin networks are sufficient to produce cooperative and telescopic contractility

**DOI:** 10.1038/ncomms12615

**Published:** 2016-08-25

**Authors:** Ian Linsmeier, Shiladitya Banerjee, Patrick W. Oakes, Wonyeong Jung, Taeyoon Kim, Michael P. Murrell

**Affiliations:** 1 Department of Biomedical Engineering, Yale University, 55 Prospect Street, New Haven, Connecticut 06520, USA; 2 Systems Biology Institute, Yale University, 850 West Campus Drive, West Haven, Connecticut 06516, USA; 3 James Franck Institute, University of Chicago, Chicago, Illinois 60637, USA; 4 School of Mechanical Engineering, 585 Purdue Mall, Purdue University, West Lafayette, Indiana 47907, USA; 5 Weldon School of Biomedical Engineering, 206 S Martin Jischke Drive, Purdue University, West Lafayette, Indiana 47907, USA

## Abstract

While the molecular interactions between individual myosin motors and F-actin are well established, the relationship between F-actin organization and actomyosin forces remains poorly understood. Here we explore the accumulation of myosin-induced stresses within a two-dimensional biomimetic model of the disordered actomyosin cytoskeleton, where myosin activity is controlled spatiotemporally using light. By controlling the geometry and the duration of myosin activation, we show that contraction of disordered actin networks is highly cooperative, telescopic with the activation size, and capable of generating non-uniform patterns of mechanical stress. We quantitatively reproduce these collective biomimetic properties using an isotropic active gel model of the actomyosin cytoskeleton, and explore the physical origins of telescopic contractility in disordered networks using agent-based simulations.

## Abstract (web summary)

The interaction between myosin motors and F-actin is well described, but the impact of actin organization on contractility is not well described. Here the authors use a 2D biomimetic system and computational modelling to show that contractility of isotropic actomyosin is cooperative, and contraction velocity scales with myosin activation area.

## Graphical abstract

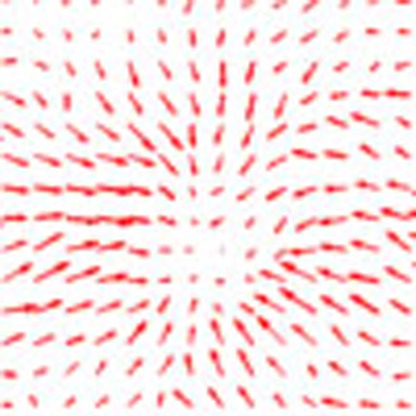

The generation of mechanical forces in cells is mediated by the diverse architectures of the actomyosin cytoskeleton^1^. In non-adherent cells, the cytoskeleton is organized into the cortex, a thin disordered F-actin network beneath the plasma membrane^2^. The cytoskeleton in adherent cells also includes stress fibres which are spatially and temporally organized across the cell^3^. Stress fibres couple the actomyosin network to the extracellular matrix (ECM) via adhesions, and are thought to be the main contractile elements in the network^4^,^5^. Myosin-driven contractility produces retrograde flow of the cytoskeletal network from the cell edge towards the cell centre. As a result, the traction stresses applied to the ECM accumulate at the cell periphery^6^,^7^, and the total work done by the cell scales with the spread area^8^,^9^,^10^,^11^. While this pattern of stress distribution is typically thought to be dependent upon the activity of stress fibres, previous reports have suggested that large traction stresses can be produced in the absence of a highly aligned and organized F-actin cytoskeleton^12^,^13^,^14^,^15^. Furthermore, physical models have proposed that disordered actomyosin networks contract^16^,^17^,^18^ and are sufficient to quantitatively reproduce the pattern of traction stresses observed in cells^11^,^19^. Thus, as F-actin organization is poorly manipulated in living cells, it is unclear how the organization of the F-actin cytoskeleton relates to the magnitude or dynamics of actomyosin contractility and stress generation.

*In vitro* model systems reconstituted from purified proteins have become a paradigm for studying cytoskeletal dynamics by enabling control over cytoskeletal organization and composition^20^,^21^,^22^,^23^,^24^,^25^,^26^,^27^,^28^. As non-equilibrium active matter^29^, these materials display novel collective dynamics including spontaneous flow, swirling waves, self-healing and topological defect dynamics^25^,^27^,^30^,^31^. However, comparing the dynamics of *in vitro* networks with the dynamics of the cell cytoskeleton has proved difficult, due to limited *in vitro* spatiotemporal control of activity. While cells precisely coordinate their mechanical activity in space and time, *in vitro* networks have so far been uniformly active.

To explore the accumulation and transmission of myosin-induced mechanical stresses in disordered F-actin networks, we have developed a novel assay (Methods) where myosin II activity is controlled in space and time within a previously established biomimetic model of the cell cortex (Fig. 1a,b)^28^,^32^. In our model, the actomyosin cytoskeleton is disordered in F-actin polarity, orientation and length (Fig. 1c–e). By altering the area of myosin activation with light from 25 μm^2^ to over 1,000 μm^2^, we can quantitatively measure the structure and dynamics of F-actin contraction from subcellular to cellular length scales (Fig. 1f). We find that contractility is highly cooperative, that the velocity of contraction scales with the size of the region of activation (that is, behaves telescopically), and that the peak velocity is predominantly localized at the periphery of the active region. Using mechanical force balance and exploiting the radial symmetry of contraction, we can calculate the spatial distribution of contractile stresses. We show that the resultant pattern of stress generation is consistent with that measured in contractile cells and cell layers. By developing a minimal mechanical model of disordered actomyosin as a viscoelastic active gel, we further show that the observed pattern of contraction arises from a trade-off between active and viscoelastic stresses in the actomyosin network. Using agent-based simulations, we then explore the molecular mechanisms that underlie F-actin contractility in disordered networks. Together our experimental data and theoretical/computational modelling establish that disordered F-actin networks alone can qualitatively reproduce the dynamics of contractile behaviour observed in cells.

## Results

### Uniform myosin activation generates localized flows

Upon illumination with 405 nm light, myosin activity initiates F-actin flow within the activation region (Fig. 2a, Supplementary Movie 1), whose direction and magnitude is quantified by measuring the F-actin velocity field (Fig. 2b, Methods and Supplementary Methods). The divergence of the velocity field becomes negative and reaches a minimum, as the network becomes maximally contractile (Fig. 2c). The divergence subsequently increases as the network aggregates as described previously^32^. Thus, the time from initial myosin activation to the minimum in the divergence characterizes the time of network contraction^28^, as opposed to the coalescence of aggregates^23^.

The F-actin flow profile changes in both magnitude and spatial distribution during contraction. The initial F-actin velocity is equally radial and tangential, but within 30 s of myosin activation, it becomes predominantly radial (Fig. 2c, inset). Simultaneously, the F-actin flow changes across the activation region from an initially uniform spatial profile to being localized at the boundary of the activation area (*r*=*ξ*) at later times (Fig. 2d). After 30 s the divergence continues to decrease, indicating that the direction of F-actin flow is stabilized quickly, whereas its magnitude continues to increase over longer timescales. Consistent with the radial flow at long times, there is increased radial alignment and nominal bundling of F-actin (Supplementary Fig. 1).

By making two simple assumptions, we estimate the spatial distribution of mechanical stresses in the actomyosin network based on the F-actin velocity profile during contraction (Supplementary Methods). First, we assume that the gel is overdamped and exerts frictional forces on the substrate. Second, we assume that the F-actin deformations, as measured by the flow field, are radially symmetric, and there is negligible azimuthal dependence (Fig. 2c, inset). We make this assumption after ∼60 s (and within 300 s), once the flow has become pronounced at the boundaries and tangential flows are suppressed. For non-circular geometries, there are elevated tangential flows, and these approximations cannot be made (Supplementary Fig. 2). Thus, without assuming any mechanical properties of the network, the predicted distribution of radial stress is found to be largest at the centre of the activation region and decays outwards towards the boundary (Fig. 2e)^33^,^34^,^35^.

### Actomyosin contraction is highly cooperative

To define the spatial extent and the dynamics of contraction, we evaluate the motor-induced strain (*ɛ*) and the strain rate (d*ɛ*/d*t*) within the activated region (Supplementary Methods). This yields a strain time course *ɛ*(*t*), which can generally be subdivided into three phases: an initial lag phase, P1 (0–100 s), a linear phase, P2 (100–300 s) and a plateau phase, P3 (>300 s) (Fig. 3a). In P1, the velocity has a significant tangential component (Fig. 2c, inset), and is therefore ignored. In P2, the strain is approximately linear with time, and represents the condensation of the disordered F-actin network. In P3, the majority of the actomyosin network has aggregated, and thus is nearing the completion of contraction. Therefore, we use the slope of the strain curve in P2 to define the strain rate in both the experiment and the model data, and take the plateau in P3 as the maximum strain value, *ɛ*_max_ (Fig. 3a, Supplementary Fig. 3).

We calculate (d*ɛ*/d*t*) and *ɛ*_max_ for a wide range of myosin densities, *ρ* (Fig. 3b,c). While we find a linear relationship between initial myosin density and the strain rate (Fig. 3b), the relationship between initial myosin thick filament density and *ɛ*_max_ displays a highly cooperative behaviour (Fig. 3c). Above a density *ρ*_c_=0.56 μm^−2^, the network contracts with a high strain magnitude (*ɛ*_max_>1) and that below *ρ*_c_, the network reaches a low level of strain (*ɛ*_max_<0.5). Strains can increase beyond one as they represent deformations in both radial and orthoradial directions, and are augmented by the flow of additional F-actin into the activation region. To calculate the cooperativity of this non-linear behaviour, we regress this data against the Hill equation (Methods), and find an extraordinarily large Hill coefficient *n*_H_=11. This behaviour is consistent with previous reports using skeletal muscle myosin that imply there is a critical stress needed to induce contraction at a fixed F-actin concentration^32^. A critical threshold of myosin concentration may also suggest a transition in the percolation of actomyosin forces for *ρ*>*ρ*_c_. Hereafter, we only consider data under conditions where *ρ* exceeds *ρ*_c_.

### Disordered actomyosin contraction is telescopic with size

For *ρ* above *ρ*_c_, we measured how the myosin-induced F-actin network strain and the strain rate varied with the radius of the activation region (Fig. 4a). While maintaining a myosin concentration above the critical threshold for contraction, we alter the total amount of active actomyosin by controlling the radius of the illuminated region. In doing so, we activate regions of three different circular sizes (mean/s.d.): small (*r*=9.0±1.6 μm, *N*=9), medium (*r*=23.1±2.6 μm, *N*=11) and large (*r*=37.1±3.2 μm, *N*=4) (Fig. 4b). We find that with increasing activation radii, there is a decrease in strain from small to medium size, but no significant changes in the magnitudes of strain or strain rate from medium to large (Fig. 4c,d). However, contraction velocity at the boundary of the activation region, calculated both by PIV (Fig. 4e) and kymograph analysis (Supplementary Fig. 4), increases with the activation radius (Supplementary Movie 2). The dependence of the velocity on the size of the activation radius is similar to the ‘telescopic' behaviour observed in bundles, where contractile velocity scales with bundle length^36^.

### Models of actomyosin network reproduce telescopic behaviour

To elucidate the physical origins of telescopic contractility, we developed a minimal mechanical model of disordered actomyosin as an active viscoelastic gel characterized by a Young's modulus *E* and viscosity *η* (ref. 37), (Methods, Supplementary Notes 1 and 2). An inner domain of size *ξ* is populated with active stresses (*σ*_a_) within an ambient passive viscoelastic medium (Fig. 5a). The composite active medium interacts with the surface underneath through a friction coefficient, *ζ*. The time-dependent flows in the network (Fig. 5b) and the accumulation of mechanical stresses (Fig. 5c) and strains are determined by the interplay between short time viscous dissipation and the rate of active stress buildup (Supplementary Notes 1 and 2). The long time behaviour is determined by network elasticity as strain reaches a plateau (Fig. 3a, Supplementary Fig. 5) and recoils if broken (Supplementary Fig. 6). The model quantitatively reproduces the strain, strain-rate and boundary velocity as observed in the experiment when the active stresses are comparable in magnitude with the gel elastic modulus. The model predicts that the maximum strain (*ɛ*_max_) and the strain rate (d*ɛ*/d*t*), spatially averaged over the activation zone, remain essentially constant, exhibiting a nominal decrease, while the maximum boundary velocity increases strongly with the size of the activation region (Fig. 5d–f), in agreement with our experimental data (Fig. 4). Varying both network elasticity (Fig. 5d–f) and network viscosity (Supplementary Fig. 7) alter the slope of the relationship between activation size and velocity. Our model suggests that stiffening (increasing *E* relative to *σ*_a_) the network can attenuate the increase in velocity with the total active motor content, although the linear relationship remains. Through F-actin crosslinking by Filamin-A (*R*_C_=[filamin]/[actin]=0.035), we bundle F-actin, and thereby increase the stiffness of the F-actin network^38^ (Fig. 5g), we indeed see that the magnitude of the average strain (Fig. 5h) and strain rate (Fig. 5i) are decreased.

As F-actin bending has been attributed to underlying contractility in disordered assemblies^18^,^28^, we can use our agent-based model to test how bending influences telescopic behaviour (Fig. 6, Methods, Supplementary Notes 3 and 4). Within the activation region, myosin motors have strong load dependence (Supplementary Fig. 8), while outside the activation area the myosin molecules are non-processive. Thus, mechanical stresses are generated locally within the activation region and propagate outside the activation area (Supplementary Movie 3), consistent with our continuum model (Fig. 5, Supplementary Figs 5, 7 and 9). For F-actin filaments with persistence length of 20 μm (Fig. 6a), the contraction at a critical myosin density and continuous increase in strain rate with myosin density are consistent with experiment (Fig. 3a–c insets and Supplementary Fig. 3). Furthermore, contraction in the simulation quantitatively reproduces the magnitude of the network strain, strain rate, and boundary velocity distribution as observed in the experiment and predicted by the continuum model (Fig. 6b–d and Supplementary Fig. 5). By contrast, for a persistence length of 2.6 mm, the increase in strain is significantly slower (Fig. 6b), which leads to much lower maximum strain rate (Fig. 6c) as well as boundary velocity (Fig. 6d). However, the increase in velocity with the size of activation region remains, suggesting that telescopic contraction is not influenced by F-actin bending but by the total amount of active actomyosin.

## Discussion

Many variables have been explored to identify the mechanisms that govern how individual cells generate mechanical forces and exert them on the ECM. Recently, it has recently been shown that the total contractile work done by the cell is principally governed by spread area^11^. Previous work, however, has suggested that the organization of the cytoskeleton plays an important role in regulating the distribution and magnitude of traction stresses^6^,^7^,^39^,^40^,^41^. This conclusion stems from observations that stress fibres contract^42^,^43^,^44^, are subject to large tensions^45^, and broadly co-localize with cellular traction stresses^6^,^46^. Furthermore, it has been shown that akin to highly organized sarcomeres *in vivo*, apolar bundles *in vitro* exhibit telescopic contraction where contractile velocity increases with bundle length^36^. However, it remained unclear whether F-actin bundling and alignment is a prerequisite for this behaviour, as cortical actomyosin, which is disordered in F-actin length, orientation and polarity, is also contractile.

In this study, we have shown that myosin activity within disordered F-actin networks is highly cooperative, as it reaches a plateau in strain with myosin concentration (Fig. 3c), indicating a possible transition in network connectivity leading to force percolation. Previous works have established that myosin activity is determined by F-actin organization^47^, and that F-actin macroscopic connectivity can be mediated by passive F-actin crosslinking proteins^22^,^48^,^49^ and myosin activity^50^. Furthermore, it has been shown that there is a relationship between the extent and rate of contractility with F-actin network connectivity^51^. Consistent with these studies, we suggest that myosin crosslinking at high myosin density (Supplementary Fig. 10) is sufficient to drive the transition in percolation and promote contractility on cellular length scales. On small length scales however, the activation radius is comparable to the mean filament length, and thus myosin crosslinking may further elevate the connectivity of the network, leading to large strains (Fig. 4c). Above this transition in connectivity, the network achieves a constant strain and the resultant patterns of internal mechanical stress are consistent with those measured in adherent cells in both their spatial distribution (Fig. 2e) and dependence on the network size (Fig. 4e).

Using a minimal continuum model of isotropic and uniformly contractile actomyosin, we show that homogeneous viscoelasticity is sufficient to reproduce the telescopic contractile dynamics observed experimentally. Our minimal coarse-grained description ignores microscale anisotropies in deformations that are evident from our experimental data (Supplementary Fig. 11). The predicted velocity profile (Fig. 5a) is thus not in perfect agreement with the experimentally measured flow profile (Fig. 2b), especially near the activation boundary, where we observe moderate shear deformations (Supplementary Fig. 12). A viscoelastic solid model was chosen to match the experimental observation that strain increases rapidly over short times and reaches a plateau at long times (Fig. 3a, Supplementary Fig. 5). In the active gel model, the dynamics of contractility are determined by the relative magnitudes of the viscoelastic and the active stress content. When balanced, the elevated velocity at the boundary arises from a sharp gradient in active contractile stress, which increases with the size of the contractile domain, and is robust to modulation of bulk mechanical parameters. However, it is likely that the mechanical properties of the actomyosin network, such as elastic modulus and viscosity, are modulated by actin density. By analysing a model where elastic modulus and viscosity are functions of density (Supplementary Fig. 9, Supplementary Note 5), we find that the telescopic behaviour is again robust to perturbations in both bulk mechanical properties and the size of the activation domain. In addition, like the linear model, increasing stiffness reduces the decrease in strain and strain rate with activation size.

As F-actin buckling has been previously implied in facilitating actomyosin network contractility^17^,^18^, we sought to determine its role in the cooperative and telescopic contractile behaviour. Increasing the persistence length of F-actin reduces its bending during contraction, thereby resisting contraction. As a result, the maximum strain rate and telescopic boundary velocity are reduced compared with those of cases with normal persistence length. However, the telescopic relationship between velocity and activation size is retained and invariant to changes in filament length (Supplementary Fig. 13). We interpret this delay as the additional time needed for myosin motors to accumulate and position themselves to generate sufficient forces that contract the network. Thus, while F-actin bending affects the rate of contraction, the network again retains its telescopic character. Taken together, the relationship between activation size and boundary velocity emerges from the accumulation of active stresses in a viscoelastic medium.

The finding that disordered actomyosin networks produce cooperative, telescopic contractility illustrates that the organization of the cytoskeleton into spatially controlled architectures, such as stress fibres, may not be required for generating this behaviour in cells. It is possible, however, that stress fibre architectures could be playing an alternative role in the actomyosin network, such as regulating the distribution of contractile stresses to the ECM. In the future, elucidating the dynamics of anisotropic and asymmetric activation will contribute to a more comprehensive understanding of the spatial regulation of cellular contractility. Our present work demonstrates that disordered actomyosin networks are independently capable of reproducing highly cooperative telescopic contractility.

## Methods

### Assay development

**Figure 1:**
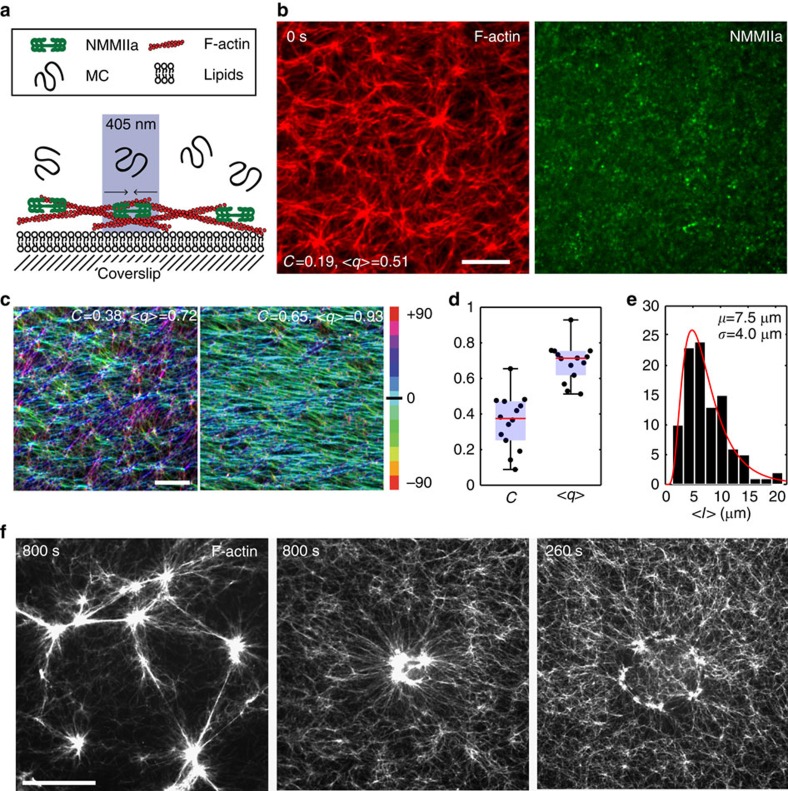
Blebbistatin inactivation promotes spatiotemporal control of myosin motor activity and F-actin network contractility. (**a**) Stabilized F-actin (red), crowded onto a lipid bilayer by methylcellulose (MC), is decorated with myosin (green), which is inhibited by blebbistatin. Light (405 nm) activates myosin activity (arrows). (**b**) Images of actin (left), non-muscle myosin II (right) embedded in 20 μM blebbistatin. Scale bar, 10 μm. (**c**) Fluorescent F-actin in blebbistatin-inhibited actomyosin networks. The colour indicates the orientation of F-actin, in two comparative cases, isotropic (left) and aligned (right). Scale bar, 10 μm. (**d**) Global coherency of F-actin orientation, *C*, and mean local nematic order parameter, *q* (*N*=13) (Supplementary Methods). (**e**) Length distribution of F-actin, *l*, prior to myosin addition (*N*=100); (**f**) contraction of F-actin network where myosin is active over entire field of view (left), blebbistatin is inactivated in a circular central region (middle) and a ring pattern (right). Scale bar, 25 μm.

**Figure 2:**
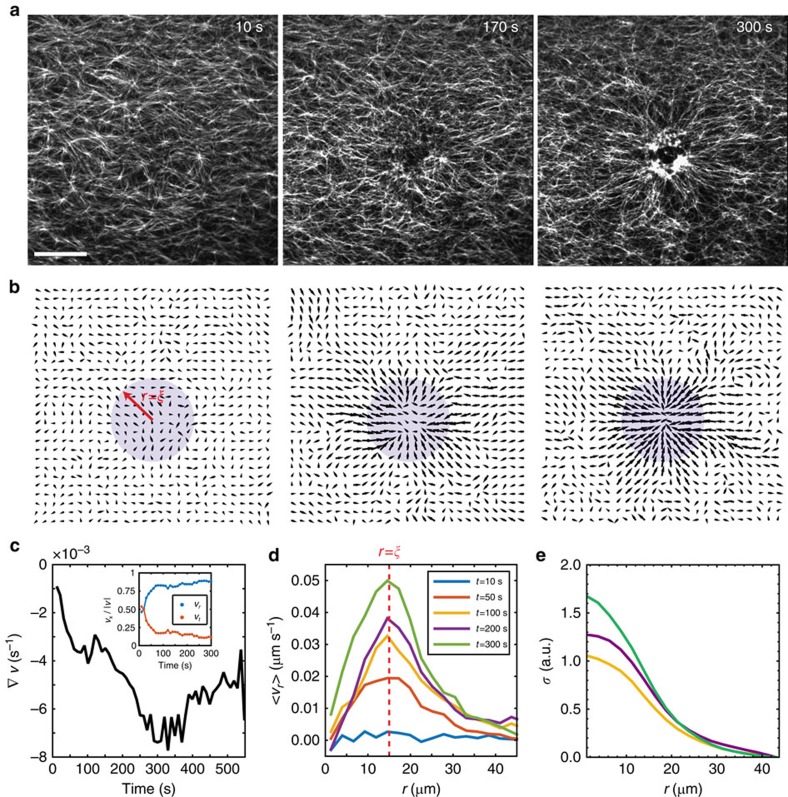
Uniform contractility yields elevated boundary flows and accumulation of active stresses. (**a**) Images of F-actin network 10, 170 and 300 s after the initial inactivation of blebbistatin. Scale bar, 20 μm. (**b**) Particle image velocimetry (PIV) of F-actin velocity field corresponding to images in **a** taken over 10 s intervals. The myosin activation area (purple) is a circular region, defined by a radius *r*=*ξ*, subject to 405 nm illumination. Vector magnitudes are normalized across all time points. (**c**) Divergence of the F-actin velocity field (d*t*=10 s) averaged over the activation area. (**c**, inset) Radial velocity (blue, *v*_r_) and tangential velocity (orange, *v*_t_) averaged over the activation area. (**d**) Mean velocity as a function of distance from the centre of the activation region, with positive values reflecting inward motion. (**e**) Predicted distribution of radial stress during contraction without assuming any materials properties of actomyosin (Supplementary Methods). Line colours correspond to timescales as indicated in **d**.

**Figure 3:**
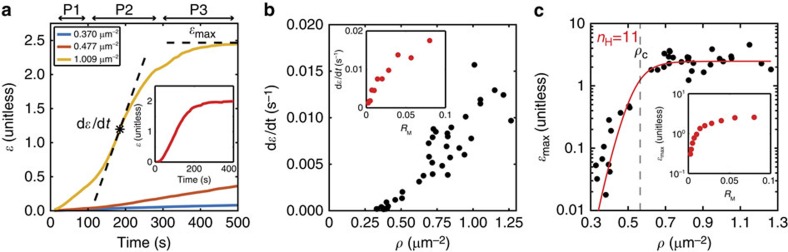
Actomyosin contractility is highly cooperative. (**a**) Network strain (*ɛ*) time course for low (*ρ*_myo_=0.37 μm^−2^) (blue), medium (*ρ*_myo_=0.48 μm^−2^) (red) and high (*ρ*_myo_=1.01 μm^−2^) (orange) myosin density. Three different stages are shown: P1 (<100 s), P2 (100–300 s) and P3 (>300 s). The strain rate, d*ɛ*/d*t* is taken at P2. The maximum network strain (*ɛ*_max_) is taken at P3. (**a**, inset) Strain at high myosin concentration in the agent-based model (*R*_M_=0.04, the ratio of myosin concentration to actin concentration). (**b**) Contractile strain rate as a function of myosin thick filament density, *ρ*. (**b**, inset) Agent-based simulation of strain rate versus *R*_M_. (**c**) Maximum contractile strain as a function of *ρ*. The red line corresponds to a fit to the Hill equation, with a hill coefficient (*n*_H_) of 11 (Supplementary Methods). The dashed line denotes the critical myosin density, *ρ*_c_=0.56 μm^−2^, above which the F-actin networks undergo significant strain (*ɛ*_max_>1). (**c**, inset) Agent-based simulation of contractile strain and *R*_M_.

**Figure 4:**
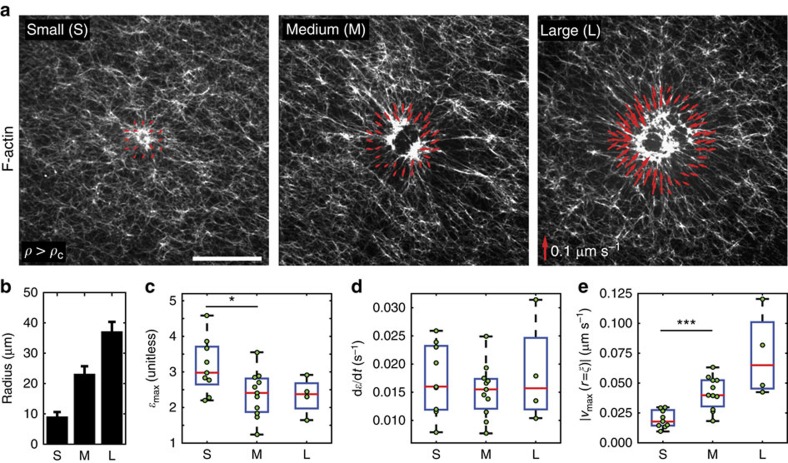
Actomyosin contraction is telescopic. (**a**) Actomyosin network contraction for activation regions of varying size: small (left), medium (middle) or large (right). Red vectors are boundary velocities at the time point of maximum contraction in each experiment. Scale bar, 25 μm. (**b**) Average radii of the activation regions. The error bars (s.d.) correspond to averaging over different illumination regions of slightly different sizes. (**c**) The maximum contractile strain (*ɛ*_max_) for each size of activation region. (**d**) Strain rate (d*ɛ*/d*t*) for activation regions. (**e**) Velocity of contraction measured at the boundary of the activation region. *P<0.05 and ***P<0.001 by a Student's *t*-test.

The development of the biomimetic model of the cell cortex has been described previously^52^. Briefly, 1.3 μM Alexa-568 labelled F-actin (Molecular Probes) is stabilized with 2 μM phalloidin (Cytoskeleton) and crowded to the surface of a 99.6% Egg Phosphatidyl Choline (Avanti Polar Lipids)/0.4% FITC-DHPE (Molecular Probes) phospholipid bilayer, using 0.25% 14,000 MW methyl-cellulose (Sigma) as a depletion agent (Fig. 1a,b). Blebbistatin (20 μM) (Sigma) is added to the sample volume prior to the addition of myosin. Following formation of the F-actin network, 24 nM of purified^53^ non-muscle myosin II (*R*_M_=0.085) labelled with Alexa-647 (Molecular Probes) is added in solution in dimeric form, which polymerizes into thick filaments onto the F-actin (Fig. 1a,b). This results in a two-dimensional actomyosin network disordered in F-actin orientation, alignment (*C*, *q*) and length *(l*) (Fig. 1c–e). Myosin density, while uniformly distributed in a given field of view (95.4 μm × 95.4 μm, Supplementary Fig. 10) at high magnification, can vary due to diffusion across the sample chamber (∼1 cm in diameter) during sample preparation^28^. A single experimental setup can therefore be used to make measurements on several different fields of view with varying myosin densities. Fields of view are always chosen more than 500 μm apart to ensure that they are mechanically isolated from each other, and the local density of myosin is determined for each field by measuring the number density of the myosin thick filaments (*ρ*, μm^−2^) present within the activation region immediately prior to light activation. Due to the presence of the blebbistatin, myosin ATPase activity is limited, thereby allowing myosin to accumulate on the F-actin network, but not induce contraction. As the network contains no F-actin crosslinking protein, all network connectivity is via myosin motors. Once the actomyosin network has been formed, we use Nikon Ti Inverted microscope equipped with a spinning disk (Yokagawa) and a Mosaic system (Photonics Instruments) to spatially inactivate blebbistatin in circular regions of interest (ROIs) via exposure to 405 nm light, thus allowing myosin to contract the F-actin network^54^,^55^. ROIs are pulsed with 405 nm light for 100 ms immediately before each image is taken of Alexa 568 labelled actin (Fig. 1f). We acquire images every 10 s, to keep blebbistatin in an inactive state within the ROI. Multiple ROIs can be taken within a single sample chamber. Imaging is performed with a × 60 1.4 numerical aperture oil immersion lens (Nikon) on a CCD Camera (Coolsnap Hq2, Photometrics).

### Analytical methods

Using Particle Image Velocimetry (PIV, Mathworks, Natick, MA), we measure the displacement vectors between individual frames at every spatial location to calculate the velocity field. By integrating the velocity field over time, we compute the local displacement field. Network Strain is then calculated by taking the divergence of the displacement field, and the strain rate is given by the divergence of the velocity field (Supplementary Methods). We also compute strain and strain rates using kymograph (ImageJ, NIH, see Supplementary Fig. 4). The boundary velocity is averaged within a 2.65 μm window about the circumference of the activation region. The filament length is calculated by tracing the fluorescence of individual F-actin in ImageJ as they are crowded to the lipid surface, and measuring the contour length.

### Active gel model

**Figure 5:**
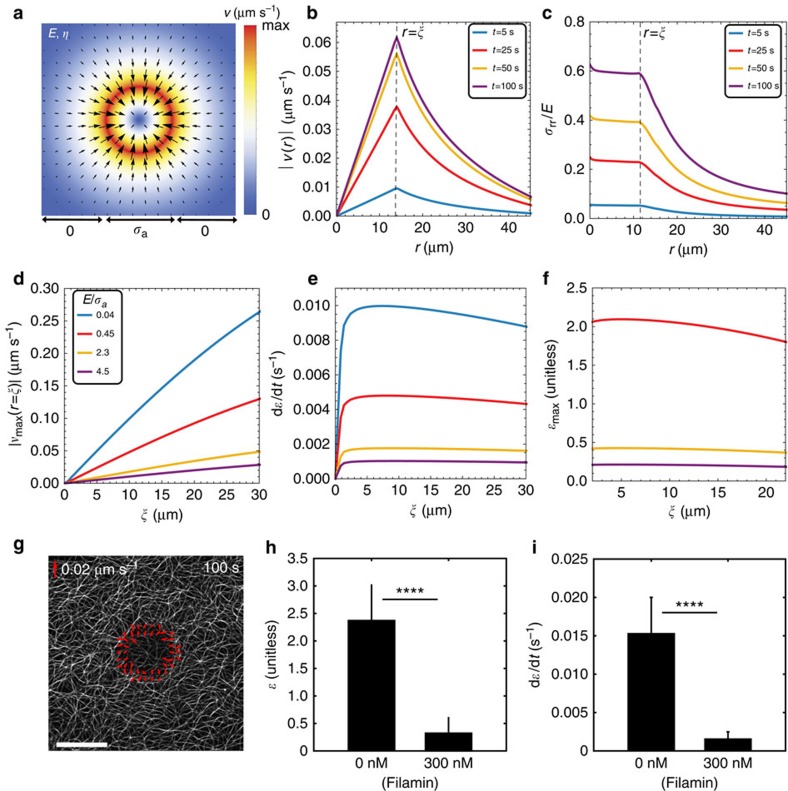
Minimal physical model of isotropic actomyosin captures telescopic contractile dynamics. (**a**) Active viscoelastic gel model of a central contractile core (with stress *σ*_a_) and surrounded by a non-contractile medium (with Young's modulus *E* and viscosity *η*). Heatmap represents magnitude of the flow velocity and arrows indicate direction of flow. (**b**) Spatial profile of radial velocity and (**c**) radial stress at different times. (**d**) Maximum velocity, (**e**) maximum strain rate and (**f**) the maximum strain as a function of the radius of the activation zone in **a** for various values of the parameter *E*/*σ*_a_. See Supplementary Table 1 for a list of model parameters. (**g**) F-actin with 45 nM Filamin-A (*R*_C_=[Filamin]/[Actin]=0.035). Red vectors indicate boundary velocity. Scale bar, 25 μm. Strain (**h**) and Strain rate (**i**) of un-crosslinked and filamin-crosslinked actomyosin. Error bars are s.d. ^****^P<0.0001 by a Student's *t*-test.

**Figure 6:**
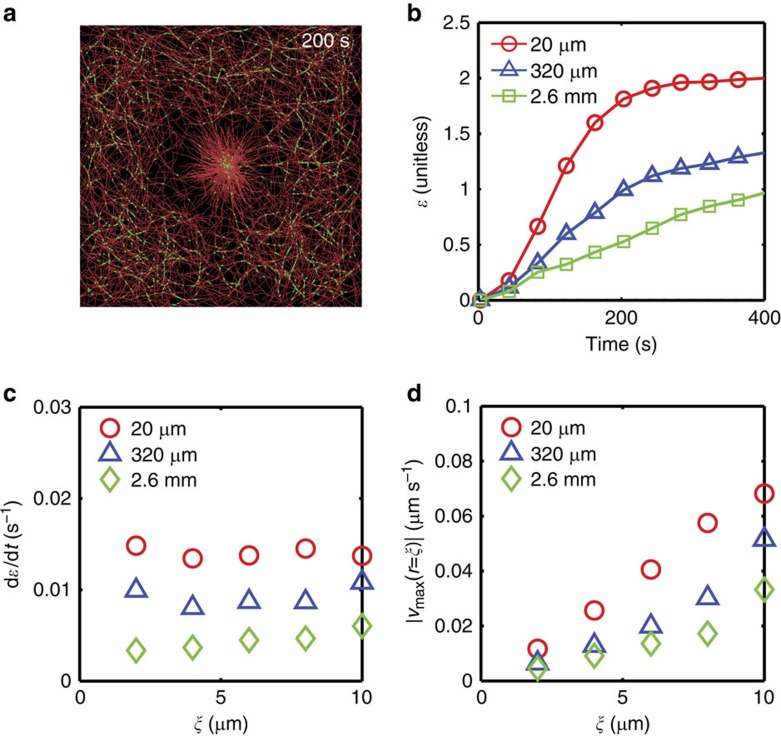
F-actin bending does not underlie telescopic contractility. (**a**) Agent-based simulation of F-actin (red) with mean and persistence length (*l*_p_) are 7.1 μm and 20 μm, respectively, and myosin motors (green) during contraction at 200 s. The width of the image is 40 μm, and the radius of the activation area is 6 μm. Myosin has lower off-rate within the activation area than outside the activation area. (**b**) Strain over time for three different persistence lengths, *l*_p_=20 μm, 320 μm and 2.6 mm. (**c**) Strain rate and (**d**) velocity as a function of the diameter of the activation region in actomyosin networks with different *l*_p_. See Supplementary Table 2 for a list of model parameters.

We describe the mechanics of disordered actomyosin using a continuum model of a homogeneous and isotropic viscoelastic gel characterized by a Young's modulus *E*, Poisson's ratio *v* and viscosity *η* (Supplementary Notes 1 and 2). The gel dynamics are overdamped and driven out of equilibrium by myosin motors that exert isotropic active contractile stresses of the form: 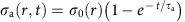, where *σ*_0_(*r*)>0 describes the spatial profile of activity and *τ*_a_ is the timescale for the accumulation of active stresses. By assuming a spatially constant active stress profile *σ*_0_ for *r*≤*ξ*, where *ξ* is the radius of the activation zone, the equations of mechanical equilibrium can be analytically solved to reproduce the observed pattern and dynamics of contraction (Fig. 5, Supplementary Notes 1 and 2).

### Agent-based model

We simulated thin actomyosin networks consisting of F-actin and active myosin motors using an approach similar to our recent works^56^,^57^ (Supplementary Note 3). F-actin is simplified as serially connected cylindrical segments. Each cylindrical segment is 280 nm in length and 7 nm in diameter, with a fixed polarity denoting the barbed and pointed ends. Motors consist of a rigid backbone with multiple arms, mimicking the geometry of myosin bipolar thick filaments^58^. The backbone consists of three cylindrical segments of 42 nm in length with symmetric polarity, connected by elastic hinges. Each endpoint of the backbone segment has two motor arms. The arms of motors can bind to binding sites located on actin segments every 7 nm, and exert force with load-dependent kinetics (Supplementary Fig. 8 and Supplementary Note 4). The mean contour length and persistence length of the F-actin is 7.1 and 20 μm.

### Data availability

The data that support the findings of this study are available from the corresponding author upon request.

## Additional information

**How to cite this article:** Linsmeier, I. *et al*. Disordered actomyosin networks are sufficient to produce cooperative and telescopic contractility. *Nat. Commun.* 7:12615 doi: 10.1038/ncomms12615 (2016).

## Supplementary Material

Supplementary Information Supplementary Figures 1-13, Supplementary Tables 1 & 2, Supplementary Notes 1-5, Supplementary Methods, Supplementary References.

Supplementary Movie 1 Light-Induced Contraction of Actomyosin Network. F-actin network contacting after a circular illumination of 405 nm light. Red vectors are flow field overlays. All displacement vectors are normalized by the maximum displacement vector magnitude over the entire timeseries. Scale bar indicates 10 μm.

Supplementary Movie 2 Contractile Velocity Increases with Activation Size. F-actin networks contracting after illumination with 405 nm light over small (left), medium (middle) and large (right) activation areas. Red vectors are flow field overlays at the boundary between active and inactive myosin. All velocity vectors are normalized across different experiments to demonstrate their relative magnitudes. Scale bar indicates 10 μm.

Supplementary Movie 3 Agent-Based Simulation of Actomyosin Contraction. (left) Agent-based simulation of F-actin (red) and myosin motors (green) whose off-rate is high outside the activation area, representing the detached state of blebbistatin-inhibited myosin, and whose off-rate is low inside the activation area, representing the myosin load-dependent kinetics of its activated state. (right) Force map of movie on left. White corresponds to filaments bearing high force, and blue filaments correspond to filaments bearing low force. Width of the image is 40 μm, and the radius of the activation area is 6 μm. Total time of simulation is 400 s.
