# Supplementary material for: Disordered actomyosin networks are sufficient to produce cooperative and telescopic contractility
Source: Nat Commun. 2016 Aug 25;7:12615. doi: 10.1038/ncomms12615 (PMC5007339; doi:10.1038/ncomms12615)
Supplement: Supplementary Information — Supplementary Figures 1-13, Supplementary Tables 1 & 2, Supplementary Notes 1-5, Supplementary Methods, Supplementary References. [file ncomms12615-s1.pdf]

Supplementary Figures

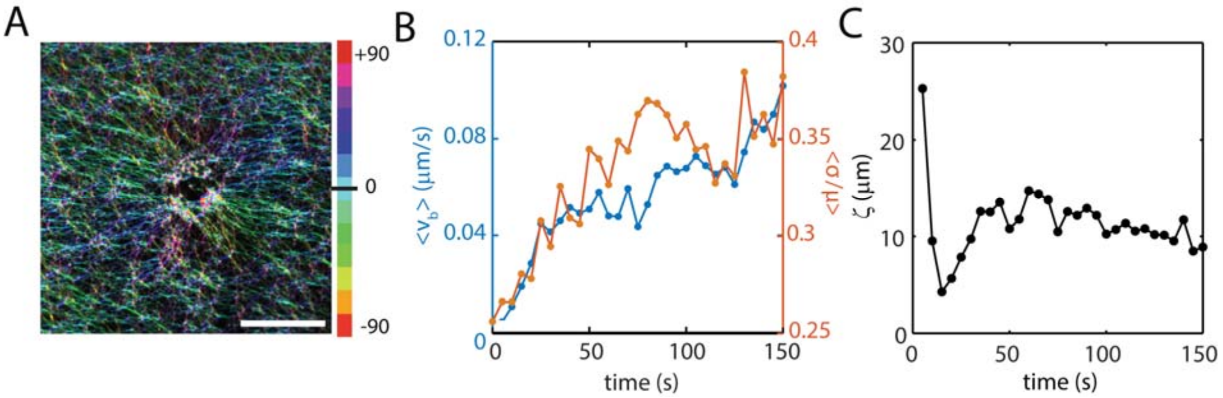

Supplementary Figure 1. Change in F-actin structure during contraction. (A) F-actin develops a radial orientation during contraction. Color indicates the direction of F-actin orientation. Scale bar is 25 µm. (B) F-actin minimally bundles, as measured by the standard deviation divided by mean in the fluorescence (orange) over circular linescans. The velocity at the boundary (blue, v_b) is linearly proportional during early times. (C) The length-scale of strain transmission (ζ) is calculated by measuring the exponential decay of the strain profile outside of the activation zone. The lengthscale is consistent with the mean filament length.

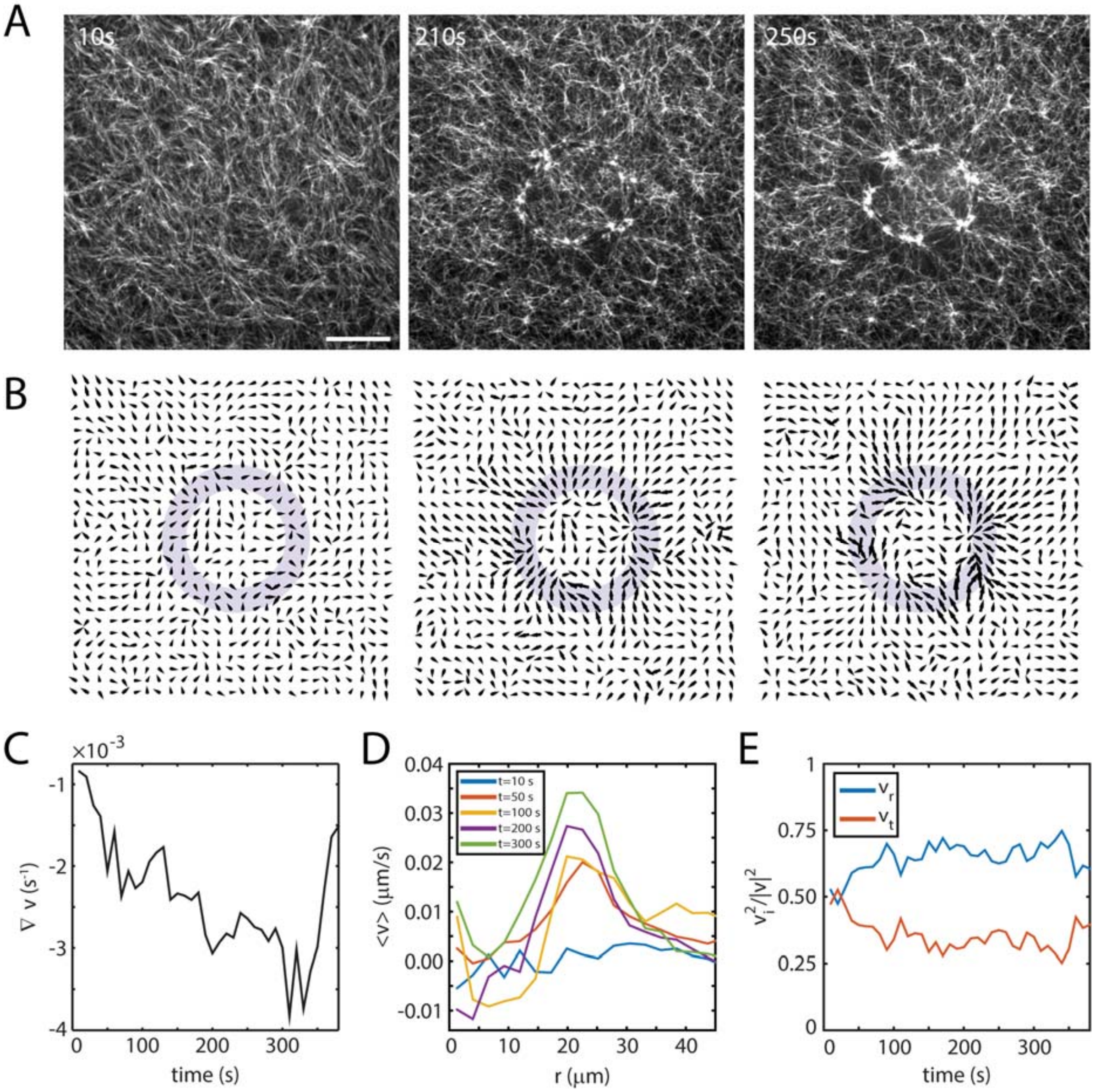

Supplementary Figure 2. Ring Activation Results in Elevated Orthogonal Flows. (A) F-actin upon 0, 220 and 260 s after illumination of 405 nm light in a ring pattern. Scale bar is 10 μm. (B) F-actin flow field after 405 nm illumination. Flow vector magnitudes are normalized over the entire time series. (C) Divergence of F-actin flow field averaged over the activation area. (D) Velocity profiles after illumination show negative velocities inside ring, positive outside ring. (E) Tangential velocities (v_t) and radial velocities (v_r) are closer when myosin is activated in ring pattern than when activated in a solid circular pattern.

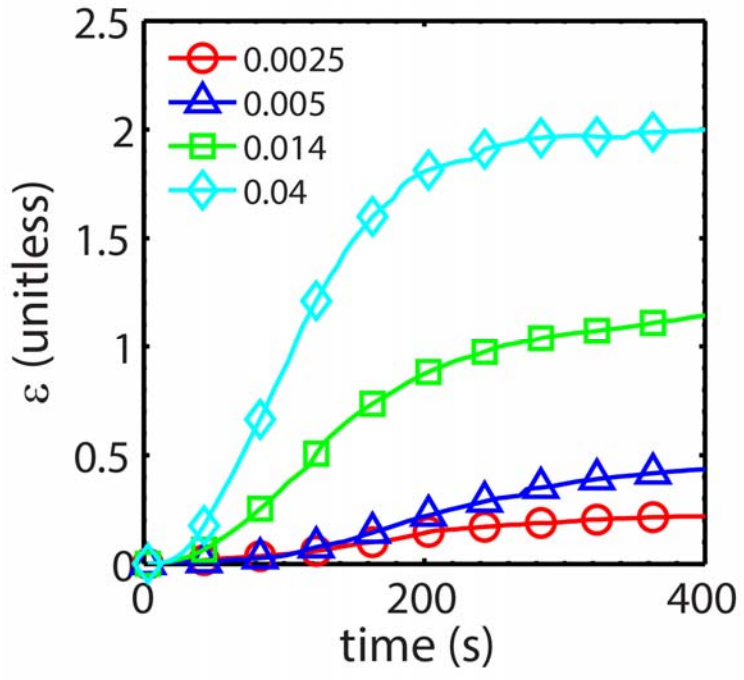

Supplementary Figure 3. Agent-based Simulation of Actomyosin Network Contraction Reproduces Strain Dependence on Myosin Density. Strain of actomyosin network, ε, at a variety of myosin densities (R_M = [myosin]/[actin]).

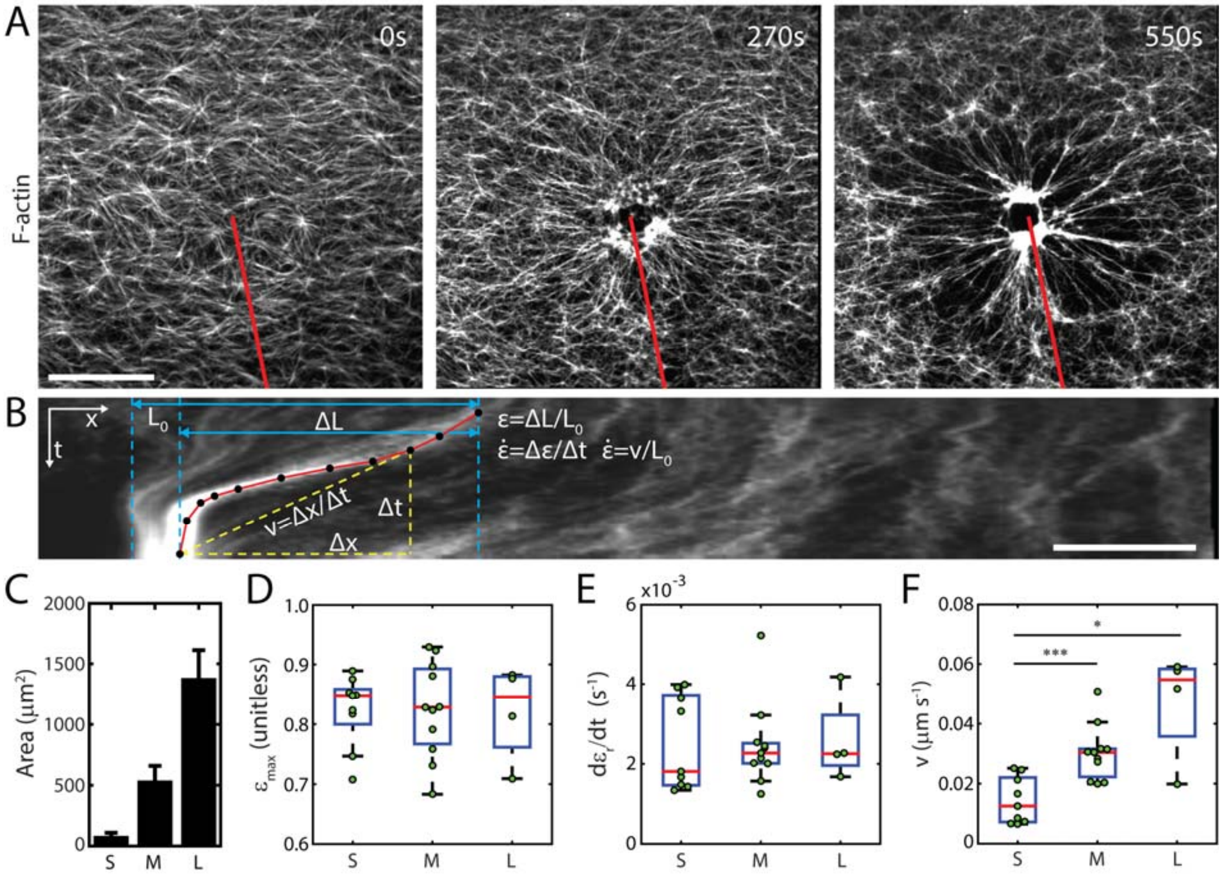

Supplementary Figure 4. Kymograph Analysis of Network Contraction Dynamics. As an alternative method to the calculation of strain, strain rate and velocity by Particle Image Velocimetry, we use kymograph analysis. (A) F-actin images 0, 270, and 550 s after the circular illumination by 405 nm light. Scale bar is 25 μm. (B) Kymograph indicating the calculation of strain and strain rate. Scale bar is 5 μm. (C) Area of illumination. Error bars are s.d. Radial strain (D), strain rate (E), and velocity (F) as calculated from the kymographs. Boundary velocity is calculated within a mean distance (ΔL) of 4.5, 8.5, and 11.4 μm from the activation boundary for the small, medium, and large regions respectively. Green dots are individual data points, bar plots represent the statistics of those points.

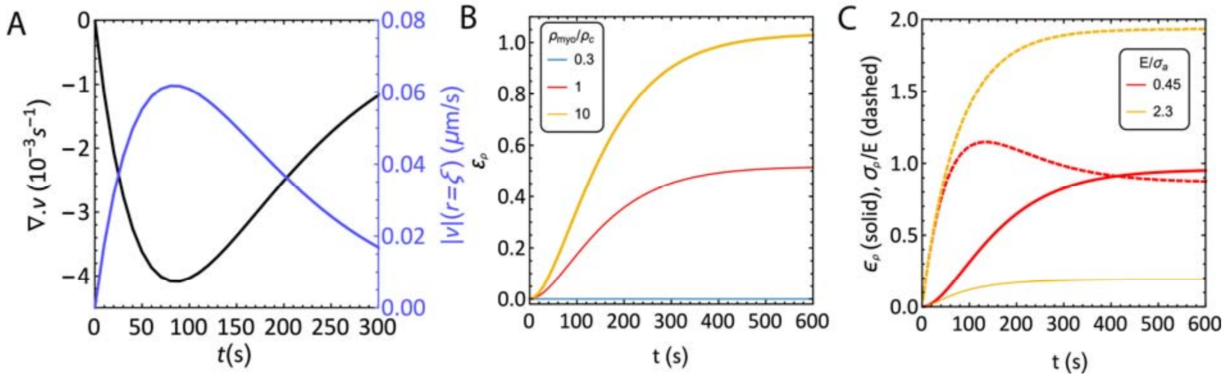

Supplementary Figure 5. Actomyosin Network Dynamics as predicted by the active gel model. (A) Contractile boundary velocity (blue) and mean velocity field divergence magnitude (black) as a function of time. (B) Actomyosin network strain (magnitude) ε_ρ, as a function of time for concentrations of myosin relative to the threshold concentration for contraction ($\rho_{\mathrm{myo}}/\rho_{\mathrm{c}}$). (C) Network strain ε_ρ(solid), and normalized stress (dashed) as a function of time for changing elastic modulus.

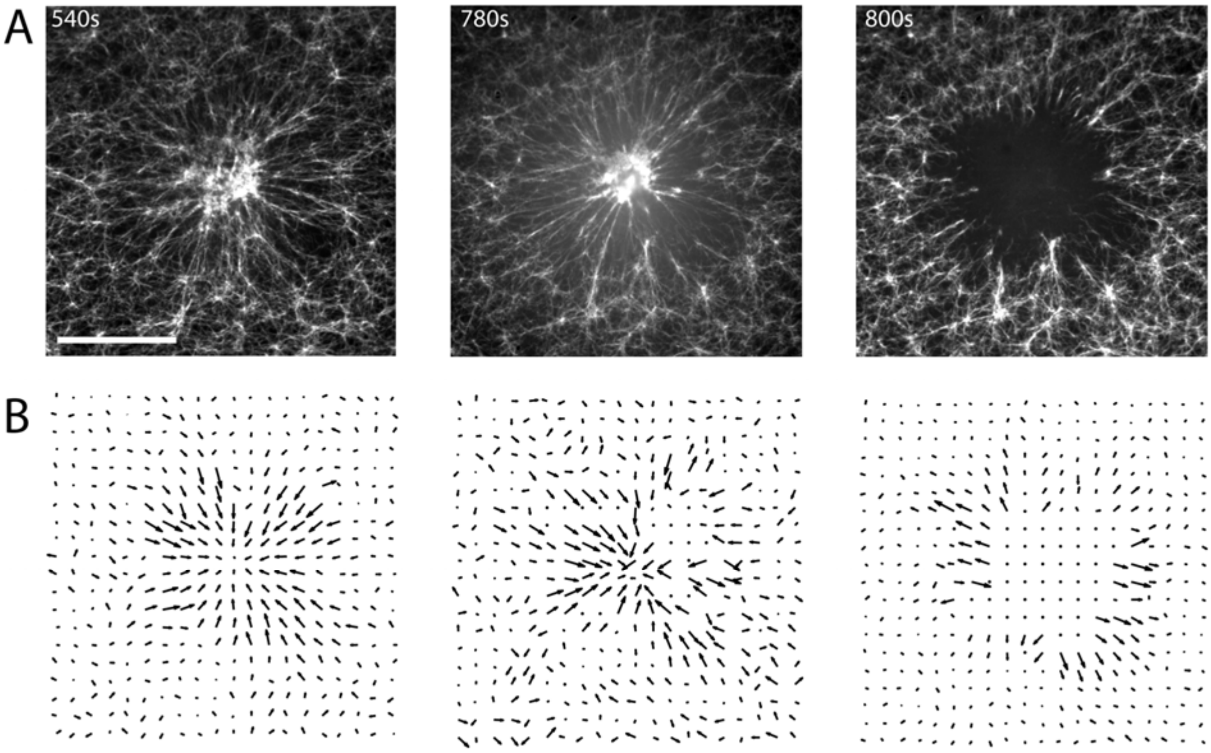

Supplementary Figure 6. F-actin Contraction is Elastic at Long Times. (A) F-actin during contraction and (B) F-actin vector field that shows inwards flow at early (left) and intermediate (middle) times, and elastic recoil (right) at long times. Scale bar is 25 μm.

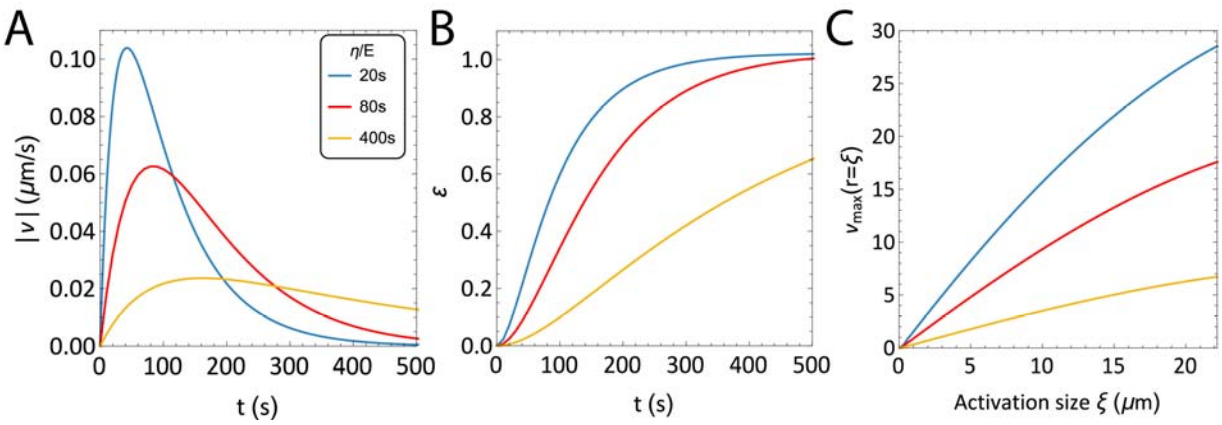

Supplementary Figure 7. Viscous Relaxation of Linear Active Gel Model. Effect of viscous relaxation timescale ($\tau=\eta/E$) on the dynamics of boundary velocity (A), radial strain (B) and the scaling of boundary velocity with activation size (C).

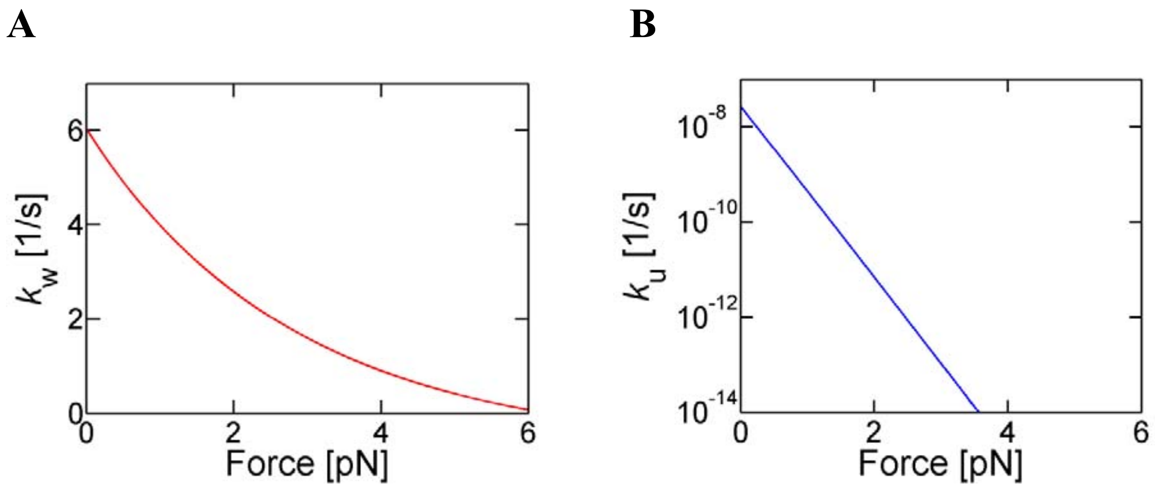

Supplementary Figure 8. Myosin Kinetics in the Agent-Based Model. Walking (k_w) and unbinding rates (k_u) of motor arms depending on force acting on the arms. They behave as a catch bond, leading to lower k_w and k_u with higher applied forces. Unloaded walking velocity is ~42 nm·s^{-1} (= 7 nm × $k_{w,M}$ at zero force), and stall force beyond which the arms stop walking is ~3.5 pN.

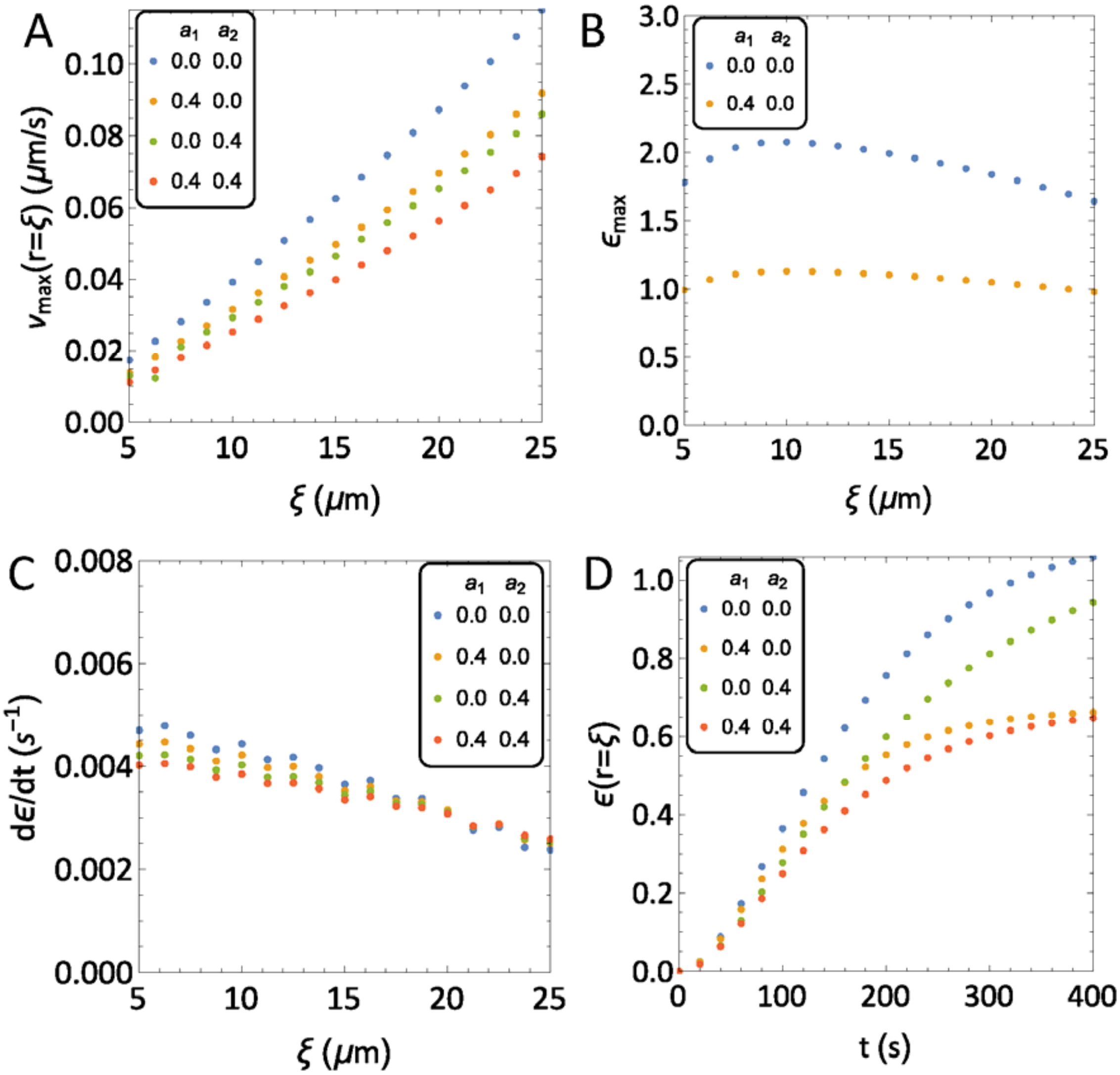

Supplementary Figure 9. Telescopic Contractility in a Non-Linear Model. In this model, $E = E_0(1 + a_1 P)$ and $\eta = \eta_0(1 + a_2 P)$, where P is the actomyosin density. (A) Dependence of edge contraction velocity vs activation size for $a_1 = 0, 0.4$ and $a_2 = 0, 0.4$. The linear relationship between boundary velocity and active domain size remains despite attentuation in telescopic contractility. (B-C) Dependence of maximum boundary strain (B) and strain-rate (C) on activation domain size. In the nonlinear model, strain and the strain-rates are more insensitive to changes in activation domain size. (D) Boundary strain vs time.

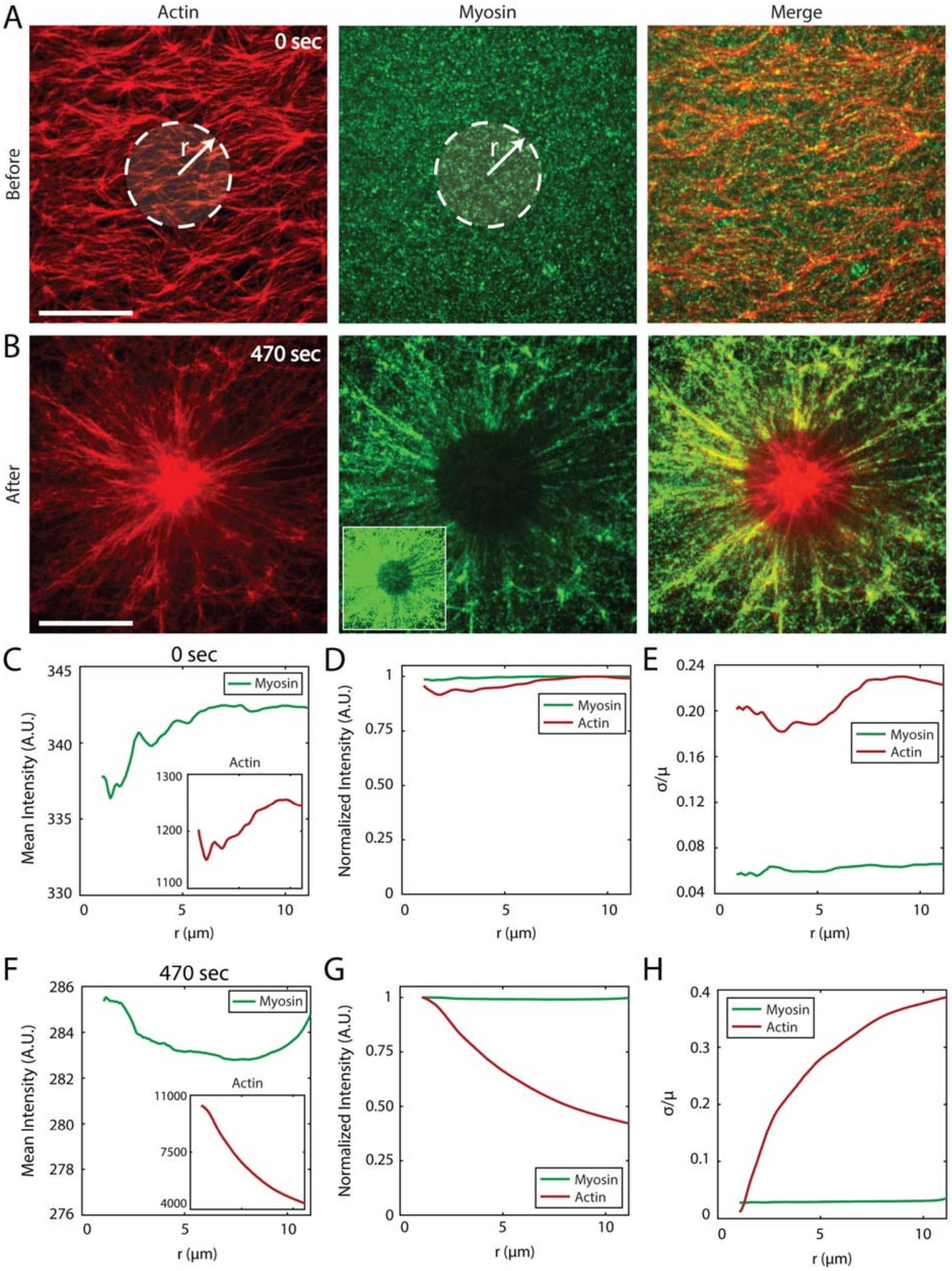
A
Actin
Myosin
Merge
0 sec
Before
r
B
470 sec
After
C
0 sec
Myosin
Actin
Mean Intensity (A.U.)
r (μm)
D
Normalized Intensity (A.U.)
Myosin
Actin
r (μm)
E
σ/μ
Myosin
Actin
r (μm)
F
470 sec
Myosin
Actin
Mean Intensity (A.U.)
r (μm)
G
Normalized Intensity (A.U.)
Myosin
Actin
r (μm)
H
σ/μ
Myosin
Actin
r (μm)

Supplementary Figure 10. Colocalization and Distribution of Myosin II before and after Contraction. (A-B) Fluoresence micrographs of F-actin (left), non-muscle myosin II (middle), and a merge the two channels (right) prior to light inactivation of blebbistatin (A) and after contraction (B). Scale bars are 20 μm. (A, overlay) The circular overlay denotes the activated region for this experiment, where *r* is the radius or boundary distance. (B, inset) Contrast enhanced micrograph of myosin to illuminate the photobleached region. (C-H) Myosin (green) and actin (red) fluoresence intensity metrics are calculated within the activation region (A, overlay) for increasing radial distances, *r*, from the contracitle center, defined as the center of the circular region. Metrics are calculated starting a small distance from the contractile center (r = 1.06 μm) up to the boundary of the activated region (r = 11.13 μm) to ensure reliable averaging. (C,F) The mean fluorescence intensity of myosin and actin (C, inset) before (C) and after (F) contraction. (D,G) The normalized fluorescence intensity of myosin and actin before (D) and after (G) contraction. (E,H) The coefficient of variation (standard deviation normalized by the mean) for myosin and actin before (E) and after (H) contraction.

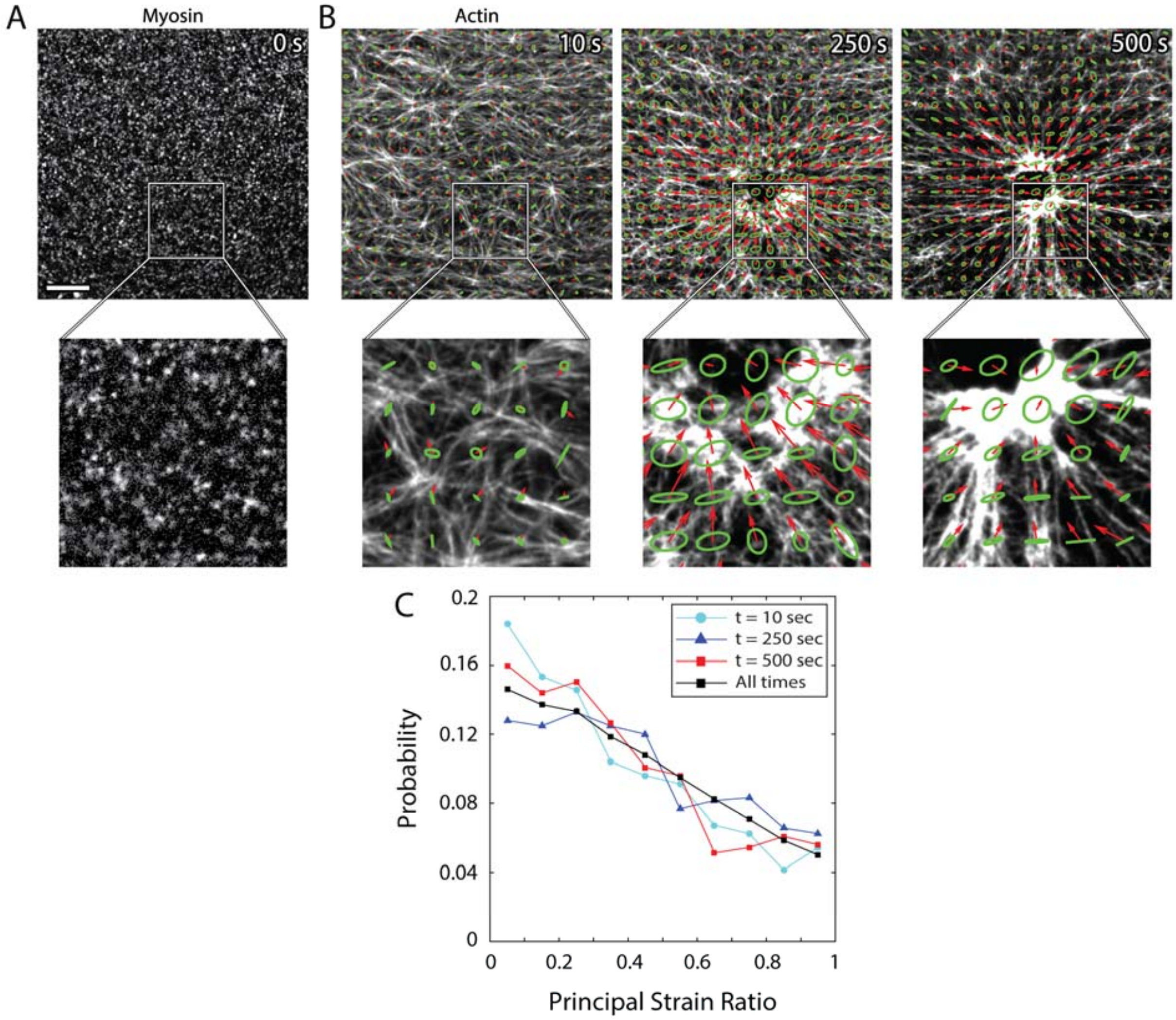

Supplementary Figure 11. Contraction is locally anisotropic. (A) Fluorescent micrograph of non-muscle myosin II thick filaments prior to contraction (t=0 s). Scale bar is 10 μm (B) Principal strain maps at t=10 s, 250 s and 500 s overlaid on the contracting actin network. The ellipses indicate the local shape of strains and the orientation reflects the direction of maximum normal strain. (C) Distribution of principal strain ratio (ratio of minimum to maximum principal strains) has a peak near zero, showing that strains are anisotropic on average (Supplementary Methods).

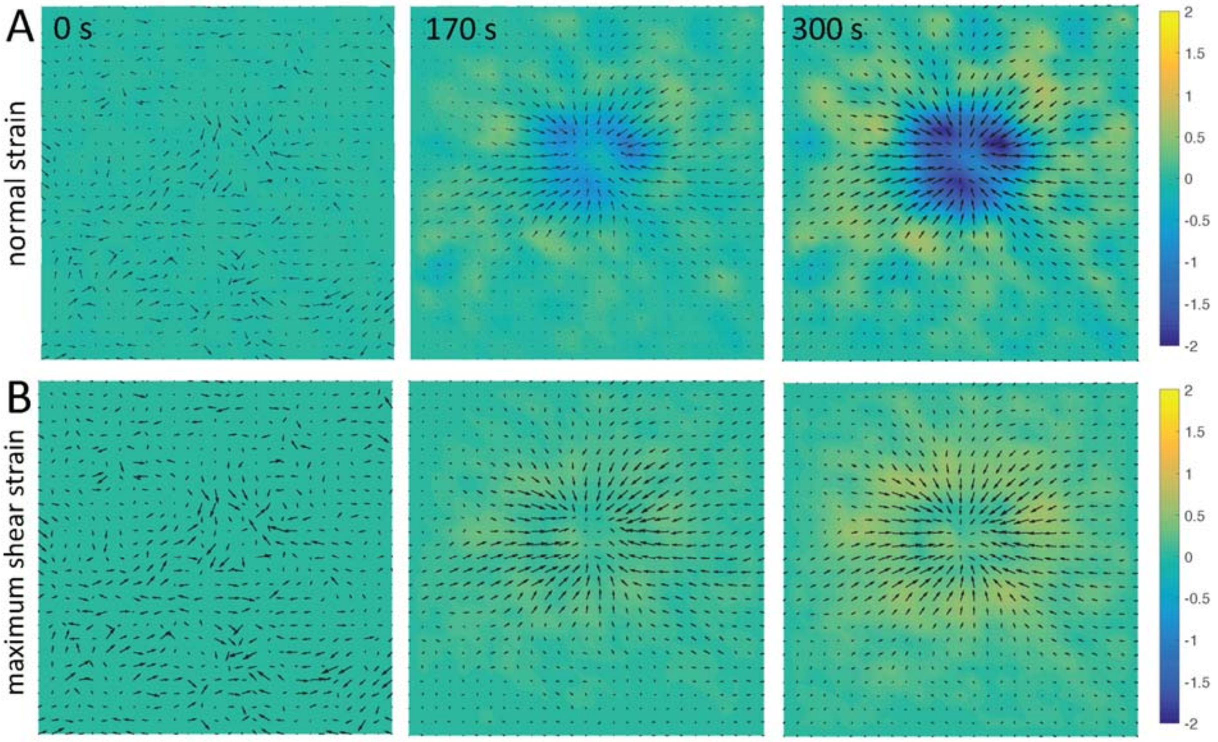

Supplementary Figure 12. Distribution of shear and normal strains during contraction. (A) Normal strain distribution at different times showing uniform contraction within the activation domain. (B) Distribution of maximum shear strain, which are lower in magnitude to normal strains, and localizes to the boundary of the activation region (Supplementary Methods).

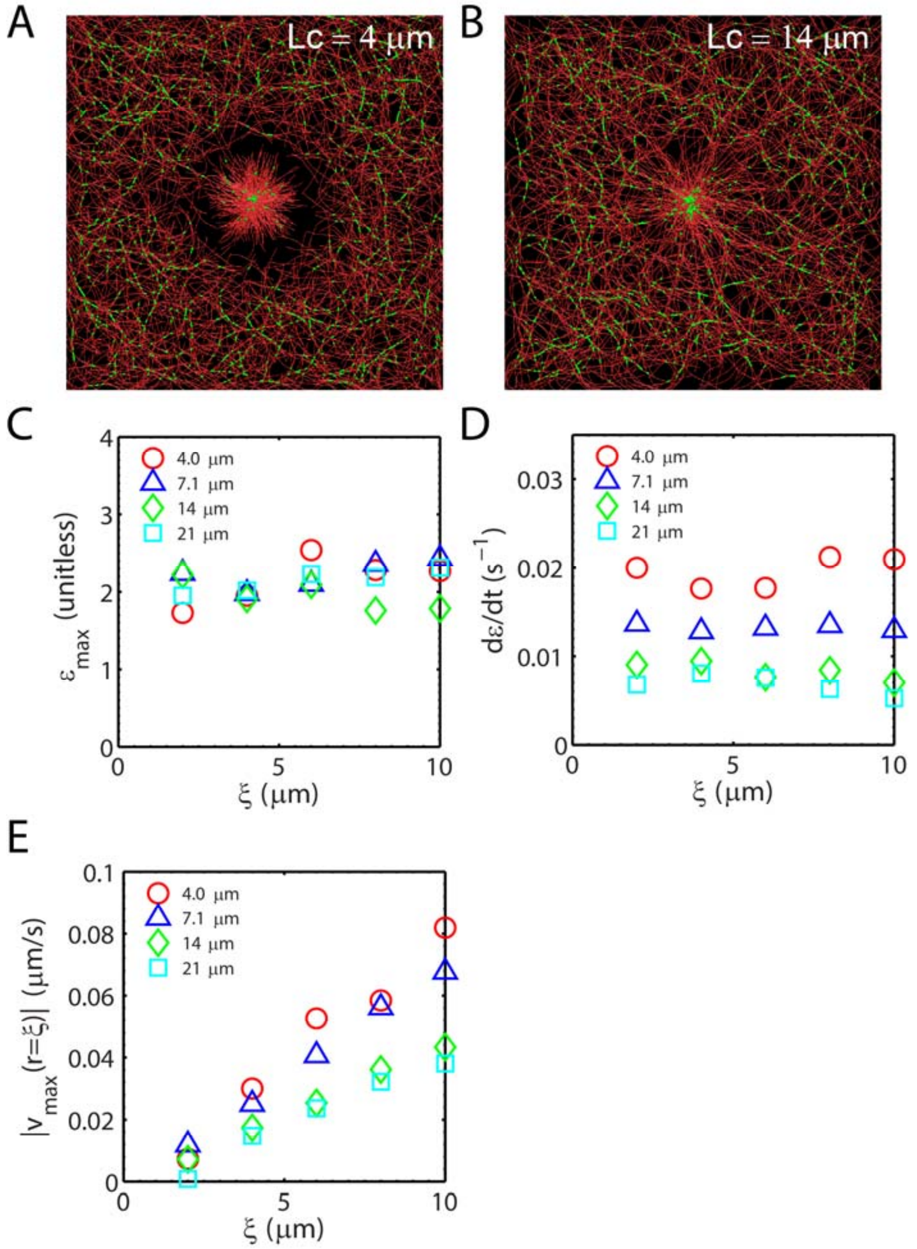

Supplementary Figure 13. Effect of F-actin length on Telescopic Contractility. Agent-based simulation of actomyosin contraction with various mean F-actin lengths (L_f). (A-B) Snapshots of networks with (A) L_c= 4 μm and (B) L_c=14 μm, taken at 200 s. Width of the image is 40 μm, and the radius of the activation area is 6 μm. (C) Maximum strain, (D) strain rate, and (E) boundary velocity of contractile actomyosin networks with varying mean filament lengths.

Supplementary Tables

Supplementary Table 1. List of parameters used in the active gel model, determined from fitting the model to the experimental data.

Parameter	Meaning	Numerical Value
τ_a	Timescale of active stress accumulation	80 [s]
$\tau = \eta / B$	Timescale of viscoelastic relaxation	85 [s]
$\tau_f = \zeta R^2 / hB$	Timescale of viscous dissipation in the substrate	200 [s]
R	System size	50×10^{-6} [m]
σ_a / B	Magnitude of active stress relative to elastic modulus	2.2 [unitless]
n_H	Myosin cooperativity constant (Hill's coefficient)	14 [unitless]

Supplementary Table 2. List of parameters used in the simulation model. Most of the parameter values are identical or very similar to those used in our recent works [12].

Symbol	Definition	Value
$r_{0,A}$	Length of an actin segment	2.8×10^{-7} [m]
$r_{c,A}$	Diameter of an actin segment	7.0×10^{-9} [m]
$\theta_{0,A}$	Bending angle formed by adjacent actin segments	0 [rad]
$\kappa_{s,A}$	Extensional stiffness of F-actin	1.06×10^{-3} [$N\cdot m^{-1}$]
$\kappa_{b,A}$	Bending stiffness of F-actin	2.97×10^{-19} [$N\cdot m$]
$r_{0,M1}$	Length of a bare zone of motor backbone	4.2×10^{-8} [m]
$r_{0,M2}$	Length of a side segment of motor backbone	4.2×10^{-8} [m]
$\theta_{0,M}$	Bending angle formed by adjacent segments constituting motor backbone	0 [rad]
$\kappa_{s,M1}$	Extensional stiffness of a bare zone	4.23×10^{-3} [$N\cdot m^{-1}$]
$\kappa_{s,M2}$	Extensional stiffness of a side segment	4.23×10^{-3} [$N\cdot m^{-1}$]
$\kappa_{b,M}$	Bending stiffness of motor backbone	5.07×10^{-18} [$N\cdot m$]
$r_{0,M3}$	Length of a motor arm	1.35×10^{-8} [m]
$r_{c,M}$	Diameter of a motor arm	1.0×10^{-8} [m]
$\kappa_{s,M3}$	Extensional stiffness 1 of a motor arm	1.0×10^{-3} [$N\cdot m^{-1}$]
$\kappa_{s,M4}$	Extensional stiffness 2 of a motor arm	1.0×10^{-3} [$N\cdot m^{-1}$]
N_h	Number of heads represented by a single motor arm	8
N_a	Number of arms per motor	8
κ_r	Strength of repulsive force	1.69×10^{-3} [$N\cdot m^{-1}$]
C_A	Actin concentration	40 [μM]
R_M	Ratio of motor concentration to C_A	0.0025-0.08
$\langle L_f \rangle$	Average length of F-actin	4.0 - 21 [μm]
Δt	Time step	4.46×10^{-5} [s]
μ	Viscosity of medium	0.86 [$kg\cdot m^{-1}\cdot s^{-1}$]
k_BT	Thermal energy	4.142×10^{-21} [J]

Supplementary Notes

Supplementary Note 1: Linear Active Gel Model

We describe a continuum model for the dynamics of disordered actomyosin based on active gel theories [15]. We assume that the thin sheet of actomyosin gel is in mechanical equilibrium with the substrate and the internal mechanics of the gel is described by a stress tensor, σ_{ij}, where i, j denote in-plane spatial coordinates. In the absence of adhesion, we assume that the substrate provides a viscous drag to the gel characterized by a friction tensor ζ_{ij}. In-plane force balance in the thin film limit gives us,

$$\zeta_{ik} v_k = h \partial_j \sigma_{ij}, \tag{S1}$$

where h is the gel thickness. Here we suggest a model for the mechanics of the gel by defining the constitutive relations for material stress.

The total stress is decomposed as the sum of elastic, dissipative and active stresses, $\sigma_{ij} = \sigma_{ij}^{el} + \sigma_{ij}^{d} + \sigma_{ij}^{a}$. While elasticity arises primarily from the compliance of F-actin filaments, dissipative stresses can arise from processes such as disentanglement of filaments and myosin binding/unbinding. Using the plane stress approximation (in the thin-film limit), the constitutive relations for elastic and dissipative stresses are given by,

$$\sigma_{ij}^{el} = \frac{E}{1+\nu}\left(\varepsilon_{ij} + \frac{\nu}{1-\nu}\varepsilon_{kk}\delta_{ij}\right), \tag{S2}$$

$$\sigma_{ij}^{d} = \eta \delta_{ij}\gamma_{kk} + 2\eta_s\left(\gamma_{ij} - \frac{1}{2}\gamma_{kk}\delta_{ij}\right), \tag{S3}$$

where $\varepsilon_{ij} = \frac{1}{2}\left(\partial_i u_j + \partial_j u_i\right)$ is the symmetrized strain tensor, $\gamma_{ij} = \partial_t \varepsilon_{ij}$ is the strain-rate tensor, E is the Young's modulus, ν is the poisson ratio, η and η_s are the bulk and shear viscosities. For simplicity we assume that myosin induced active stresses are isotropic, with the functional form,

$$\sigma_{ij}^{a} = \sigma_0 \frac{(\rho/\rho_c)^{n_H}}{1+(\rho/\rho_c)^{n_H}} \left(1 - e^{-t/\tau_a}\right) \delta_{ij}, \tag{S4}$$

where $\sigma_0 > 0$ is the magnitude of contractile stress, n_H is a positive constant (Hill coefficient), ρ is the myosin density (assumed to be uniform), ρ_c is the density beyond which myosin induced stresses saturate, and τ_a is the timescale for the accumulation of active stresses. To model the experimental geometry, we assume a spatially constant active stress profile σ_0 for $r \le \xi$ (and zero outside), where ξ is the radius of the activation zone. We now exploit radial symmetry of the flow profile as observed experimentally to assume that all the quantities are azimuthally invariant. In polar coordinates, force balance in the radial direction is given by the stress equilibrium condition (Eq. S17). In terms of the radial displacement field (u_r), the force balance equation simplifies to,

$$\frac{\zeta}{h}\partial_t u_r = \left(B + \eta\partial_t\right)\left(\partial_r^2 u_r + \frac{1}{r}\partial_r u_r - \frac{u_r}{r^2}\right) + \partial_r \sigma_a(t), \tag{S5}$$

where $B = \frac{E}{1-\nu^2}$ is the compressional elastic modulus of the gel. Eq. (S5) obeys the boundary conditions, $u_r(r=0)=0$ and $\sigma_{rr}(r=R)=0$. We expand u_r in Fourier-Bessel series as, $u_r(r,t) = \sum_{n=0}^{\infty} u_n(t) J_1(k_n r/R)$. The eigenmodes k_n are governed by the roots of the equation, $\frac{1}{1-\nu} J_0(k_n) = J_1(k_n)$. The Fourier-Bessel amplitudes, $u_n(t)$, are given by,

$$\left(\frac{\zeta}{h} + B\tau k_n^2\right)\frac{du_n}{dt} = -Bk_n^2 u_n - \frac{2\sigma_a \xi J_1(k_n \xi/R)}{J_2(k_n)^2}\left(1 - e^{t/\tau_a}\right) \tag{S6}$$

where, $\tau = \eta / B$ is the viscoelastic timescale and $\sigma_a = \sigma_0 \frac{(\rho/\rho_c)^{n_H}}{1+(\rho/\rho_c)^{n_H}}$.

The solution to (S6) can be obtained analytically using the initial condition of zero displacement (the solution is cumbersome and hence not presented here). Using the analytical solution for

radial displacement we can then evaluate mechanical quantities such as radial strain ($\varepsilon_{rr} = \partial_r u_r$), orthoradial strain ($\varepsilon_{\theta\theta} = u_r / r$), velocity ($v_r = du_r / dt$), strain-rates ($d\varepsilon_{rr} / dt$ and $d\varepsilon_{\theta\theta} / dt$) and the corresponding stresses.

Supplementary Note 2: Active Gel Model Myosin Dynamics

In our continuum model we treat actomyosin as a single unit, and assume a uniform active stress profile. The active stresses arise from myosin that are highly dynamic. The concentration dynamics of myosin minifilaments (average length *l*) bound to actin can be modeled using the following equation [16]:

$$\partial_t \rho = D\nabla^2 \rho + \nabla \cdot (\rho \partial_t \boldsymbol{u}) - k_u \rho + k_b(\rho_0 - \rho) \tag{S7}$$

where *D* is a diffusivity consant, k_u is the unbinding rate of myosin, k_b is the binding rate and ρ_0 is the total concentration of myosin. The equation tells us that density fluctuations in myosin relax over a characteristic timescale, $\tau_a = 1/(k_b + k_u + D/l^2)$, beyond which myosin is primarily advected by actin flow.

Supplementary Note 3: Agent-Based Simulation

Displacement of each cylindrical segment is governed by the Langevin equation with inertia neglected:

$$\mathbf{F}_i - \zeta_i \frac{\mathrm{d}\mathbf{r}_i}{\mathrm{d}t} + \mathbf{F}_i^{\mathrm{T}} = 0 \tag{S8}$$

where $\mathbf{r}_i$ is the position of endpoint of cylindrical segments, ζ_i is an effective drag coefficient, *t* is time, and $\mathbf{F}_i$ is a net deterministic force including extensional, bending, and repulsive forces. $\mathbf{F}_i^{\mathrm{T}}$ is a stochastic force determined by the fluctuation-dissipation theorem [6]:

$$\left\langle \mathbf{F}_i^{\mathbf{T}}(t)\mathbf{F}_j^{\mathbf{T}}(t)\right\rangle = \frac{2k_{\mathbf{B}}T\zeta_i\delta_{ij}}{\Delta t}\boldsymbol{\delta} \tag{S9}$$

where δ_{ij} is the Kronecker delta, $\boldsymbol{\delta}$ is a unit second-order tensor, and Δt is time step. For the cylindrical geometry of segments, we used the approximate form of ζ_i [7]:

$$\zeta_i = 3\pi\mu r_{c,i} \frac{3 + r_{0,i} / r_{c,i}}{5} \tag{S10}$$

where $r_{c,i}$ and $r_{0,i}$ are the diameter and length of a segment, respectively. Position of each segment is updated using the Euler integration scheme:

$$\mathbf{r}_i(t + \Delta t) = \mathbf{r}_i(t) + \frac{d\mathbf{r}_i}{dt}\Delta t = \mathbf{r}_i(t) + \frac{1}{\zeta_i}\left(\mathbf{F}_i + \mathbf{F}_i^{\mathrm{T}}\right)\Delta t \tag{S11}$$

The extension and bending of actin and motor are governed by harmonic potentials with stiffness κ_s and κ_b, respectively:

$$U_s = \frac{1}{2}\kappa_s (r - r_0)^2, \quad U_b = \frac{1}{2}\kappa_b \left(\theta - \theta_0\right)^2 \tag{S12}$$

where r is a distance, θ is a bending angle, and the subscript 0 denotes the equilibrium value. Extensional ($\kappa_{s,A}$) and bending stiffnesses of F-actin ($\kappa_{b,A}$) maintain an equilibrium length of actin segments ($r_{0,A}$ =280 nm) and an equilibrium angle formed by adjacent actin segments ($\theta_{0,A}$ = 0 rad). Similarly, the extensional ($\kappa_{s,M1}$ and $\kappa_{s,M2}$) and bending stiffnesses of motor backbone ($\kappa_{b,M}$) maintain equilibrium lengths of the backbone ($r_{s,M1}$ = $r_{s,M2}$ = 42 nm) and an equilibrium angle formed by adjacent backbone segments ($\theta_{0,M}$ = 0 rad). Extension of each motor arm is governed by a two-spring model where a transverse spring ($\kappa_{s,M3}$) maintains an equilibrium distance ($r_{0,M3}$ = 13.5 nm) between F-actin and the endpoint of the motor backbone segment while a longitudinal spring ($\kappa_{s,M4}$) maintains a right angle between axis of the F-actin and a motor arm ($r_{0,M4}$ = 0 nm). Bending and extensional forces exerted on the binding spots of an actin segment due to interactions with motors are distributed onto the barbed and pointed endpoints of the segment as described in our previous work [8].

Repulsive forces that simulate volume-exclusion effects are calculated via the following

harmonic potential [9]:

$$U_{\mathrm{r}} = \begin{cases} \frac{1}{2}\kappa_{\mathrm{r}}\left(r_{12} - r_{\mathrm{c,A}}\right)^2 & \text{if} \quad r_{12} < r_{\mathrm{c,A}} \\ 0 & \text{if} \quad r_{12} \geq r_{\mathrm{c,A}} \end{cases} \tag{S13}$$

where κ_{r} is strength of repulsive force, and r_{12} is the minimum distance between a pair of actin segments.

Each of the motor arms attached to the backbone represents 8 myosin heads (N_{h} = 8). Thus, each motor with 8 arms behaves as a thick filament with 64 myosin heads. A free arm can bind to a binding site on F-actin at a rate, $40 \cdot N_{\mathrm{h}} \cdot \mathrm{s}^{-1}$. Motor arms walk on and unbind from F-actin following the walking (k_{w}) and unbinding rates (k_{u}) which vary depending on forces applied to the arms (Supplementary Fig. 8). At each walking event, arms slide from a current binding site to a next one located toward the barbed end by ~7 nm. After reaching the barbed end, motors slide off from F-actin via a next walking event. Note that it is assumed that myosin heads behave as a catch bond [10, 11], leading to lower k_{w} and k_{u} with higher applied forces. We assume that deactivated motors located outside the activation zone unbind at a much higher rate ($10^5 \times k_{\mathrm{u}}$) and cannot walk.

We preassemble cross-linked actomyosin networks via self-assembly of actin monomers (G-actin) and motors within a thin three-dimensional rectangular domain (40×40×0.1 μm) without periodic boundary condition. All boundaries of the domain exert repulsive forces to keep network elements within the domain. During the self-assembly, G-actin are nucleated and polymerized into F-actin, and the thick filament structure of motors is formed via nucleation and polymerization of motor backbone segments with their arms binding to F-actin in the absence of walking events. Unless specified, we used the reference values for parameters: initial G-actin concentration (C_{A}) is 40 μM, average F-actin length ($<L_{\mathrm{f}}>$) is ~7.1 μm, and the molar ratio of motors ($R_{\mathrm{M}} = C_{\mathrm{M}} / C_{\mathrm{A}}$) is 0.04.

Supplementary Note 4: Agent-Based Model Abstraction

Blebbistatin binds the myosin-ADP-Pi complex, and inhibits phosphate release. Thus, it keeps myosin in the actin-detached state, preventing rigid cross-linking [13, 14] effectively reducing processivity. To this effect, in our simulations, we increase myosin unbinding in regions where blebbistatin is active and decrease myosin unbinding in regions where blebbistatin has been inactivated. In doing so, the F-actin outside the activation region is loosely crosslinked, and thus as contraction proceeds in the activation region, the F-actin separates between these two regions.

Supplementary Note 5: Nonlinear Active Gel Model

In the nonlinear active gel model we assume that the elastic modulus and the viscosity are functions of actomyosin density, P, such that

$$E = E_0(1 + a_1 P), \tag{S14}$$

$$\eta = \eta_0(1 + a_2 P), \tag{S15}$$

where a_1 and a_2 are positive constants. As a consequence, the network stiffens and becomes more viscous with increasing density. The constitutive laws for elastic and viscous stresses are assumed to be linear in strain and strain rates, respectively, as defined in Eqs. (S2) and (S3). Since changes in density are slaved to the network strain, we have the continuity equation:

$$\partial_t P = -\nabla \cdot (P \partial_t \boldsymbol{u}). \tag{S16}$$

Using this simple form for strain-dependent viscoelastic response, we numerically solve the resultant force balance equations (Mathematica, NDSolve), Eq. (S1), assuming spatially uniform contractile stress within the activation domain. By varying the parameters a_1 and a_2 in the model, we can control the strength of nonlinear coupling and density dependent variations in mechanical properties. We find that the telescopic behavior is retained for non-zero values of a_1 and a_2 (Supplementary Fig. 9A), but the slope of the relationship between velocity and

activation is reduced for networks stiffening ($a_1 > 0$) and thickening ($a_2 > 0$) with increasing density. This attenuation in telescopic contractility results from concomitant reductions in sptially averaged maximum strain and strain-rates (Supplementary Fig. 12 B,C).

Supplementary Methods

Velocity Calculations

The velocity field of the F-actin is calculated using Particle Image Velocimetry (PIV) (mPIV; www.oceanwave.jp/softwares/mpiv/). mPIV creates a regular grid across the image, where the velocity is calculated for each grid location between a pair of images. The strain is taken as the divergence of the F-actin displacement field, and the strain rate is the divergence of the velocity field, for the experiment, model and simulation. For each metric, the average is taken over the activation zone. This leads to strains larger than 2, as in addition to the sum of radial and orthoradial components, there is additional F-actin which flows into the activation region over the time at which the strain and strain rate are averaged. The velocity is averaged only within a narrow vicinity at the boundary, not throughout the activation area.

Mechanical Stress Calculation

In mechanical equilibrium, Eq. (S1) provides the condition for local force-balance in the gel. Now assuming radial symmetry, mechanical equilibrium in polar coordinates satisfies the equation,

$$\partial_r \sigma_{rr} + \frac{1}{r}\left(\sigma_{rr} - \sigma_{\theta\theta}\right) = \frac{\zeta_{rr}}{h} v_r, \qquad \text{(S17)}$$

where σ_{rr} and $\sigma_{\theta\theta}$ are the two normal stresses in the radial and the orthoradial directions. The general solution can be expressed in terms of an *Airy stress function* ϕ [4],

$$\sigma_{rr} = \frac{1}{r}\frac{\partial\phi}{\partial r} - \frac{\zeta_{rr}}{h}\int_{r}^{\infty} dr' v_r(r'), \tag{S18}$$

$$\sigma_{\theta\theta} = \frac{\partial^2\phi}{\partial r^2} - \frac{\zeta_{rr}}{h}\int_{r}^{\infty} dr' v_r(r'). \tag{S19}$$

Since ϕ is a biharmonic function, we can use the ansatz [5], $\phi = ar^2 + br^2\log(r) + c\log(r)$. Using the condition that σ_{rr} is finite at $r=0$, we have $b=0$ and $c=0$. To determine the constant a, we use stress boundary condition $\sigma_{rr}(r = R) = 0$, where R defines the total system size ($R \gg \xi$). The profile for radial stress can then be obtained from the experimentally measured flow profile, up to an undetermined multiplicative factor, $\frac{\zeta_{rr}}{h}$,

$$\sigma_{rr}(r) = -\frac{\zeta_{rr}}{h}\int_{r}^{R} dr' v_r(r'). \tag{S20}$$

Since the flow velocity points radially inward ($v_r < 0$) and localizes at the boundary of the activation region, the internal stress is positive and largest at the center of the activation region where it accumulates over time (Fig. 2E).

Principal Component Analysis

To analyze the local shape and magnitudes of strain and strain-rates we perform a principal component analysis of the symmetric strain tensor [4], defined as:

$$s_{ij} = \frac{1}{2}\left(\partial_i u_j + \partial_j u_i\right) \tag{S21}$$

where $\boldsymbol{u}$ is the displacement field. The symmetric strain-rate tensor is given by the time derivative of the strain tensor, or equivalently as the spatial gradients of the velocity field. The principal strains, s_1 and s_2, are defined by the eigenvalues of the strain tensor. Their magnitudes define the major and the minor axes of the principal strain ellipse, which characterizes the shape of local strains. For example, isotropic strains correspond to $s_1 = s_2$, whereas uniaxial

deformations imply $s_1 = 0$ or $s_2 = 0$. The orientations of the ellipse axes correspond to the eigenvectors of the strain tensor, such that the major (minor) axis points along the direction of maximum (minimum) normal strain. Total normal strain is given by $s_1 + s_2$, which equals the divergence of the displacement field. The local maximum value of the shear strain, s_{xy}, is related to the difference of the principal strains: $|s_1 - s_2|/2$ [4].

Hill Model Regression

The regression to the Hill Model (Fig 3C) was performed using Origin (OriginLab Corp). The reaction velocity is replaced with the network strain, and the substrate concentration is the 'effective concentration' of myosin ([M]), measured as the number of myosin thick filaments per unit area (μm^{-2}). Thus, V_{max}, and ε are unitless. The equation is as follows:

$$\epsilon \; = \frac{V_{max}[M]^{nH}}{(K_{0.5})^{nH} + [M]^{nH}} \tag{S22}$$

Three parameters, were fit, V_{max}, $K_{0.5}$ and n_H are as follows: 2.48, 0.56 μm^{-2}, and 10.95 respectively. The Hill coefficient represents the cooperativity of myosin behavior towards generating strain. More specifically, assuming that the density of myosin thick filaments is proportional to the total density of actomyosin bonds, the hill coefficient represents the sensitivity of the strain to the number of actomyosin bonds. V_{max} and $K_{0.5}$ represent the maximum strains accomplished during contraction, and the quantity of myosin it would take to reach half of that strain.

Orientation Quantification

Analysis of the orientation of F-actin filaments was performed using the ImageJ (NIH) plugin OrientationJ (EPFL; http://bigwww.epfl.ch/demo/orientation/). The color is orientation, the saturation is coherence, and the brightness is the original image brightness. The global coherency of F-actin is measured by C and represents the ratio between the difference and the sum of the

tensor eigenvalues, and is bounded between 0 (isotropic) and 1 (aligned) [1].

Order Parameter Calculation

The nematic order parameter, q was calculated as performed previously [2] [3] using Custom written Matlab (Mathworks, Natick, MA) routines. Briefly, the image was broken down into a series of small windows (3.5 x 3.5 μm) to determine the local orientation of filaments. For each window, a symmetric 2D Gaussian filter was applied, and then the 2D FFT was determined. The orientation of the FFT transform was determined by calculating the least second moment of rotation. The orientation in real space is orthogonal to the orientation calculated in frequency space. To calculate the order parameter, q, a given orientation vector was compared to its nearest neighbors using the following equation:

$$q = 2\left(\langle \cos^2 \theta_{ij} \rangle - 1/2\right) \quad \text{(S23)}$$

The order parameter thus calculates the degree of F-actin alignment, with 0 being unaligned, and 1 being highly aligned.

Supplementary References

1. Rezakhaniha, R., et al., *Experimental investigation of collagen waviness and orientation in the arterial adventitia using confocal laser scanning microscopy.* Biomechanics and Modeling in Mechanobiology, 2012. **11**(3-4): p. 461-473.
2. Aratyn-Schaus, Y., P.W. Oakes, and M.L. Gardel, *Dynamic and structural signatures of lamellar actomyosin force generation.* Mol Biol Cell, 2011. **22**(8): p. 1330-9.
3. Cetera, M., et al., *Epithelial rotation promotes the global alignment of contractile actin bundles during Drosophila egg chamber elongation.* Nat Commun, 2014. **5**: p. 5511.
4. Bower, A.F., *Applied mechanics of solids*. 2010, Boca Raton: CRC Press. xxv, 794 p.
5. Michell, J.H., *On the Direct Determination of Stress in an Elastic Solid, with application to*

the Theory of Plates. Proc. London Math. Soc, 1899. **s1-31**(1): p. 100-124.

6. Underhill, P.T. and P.S. Doyle, *On the coarse-graining of polymers into bead-spring chains.* J Non-Newton Fluid, 2004. **122**(1-3): p. 3-31.
7. Clift, R., J.R. Grace, and M.E. Weber, *Bubbles, drops, and particles*. 2005, Mineola, NY: Dover Publications.
8. Kim, T., *Determinants of contractile forces generated in disorganized actomyosin bundles.* Biomech Model Mechanobiol, 2015. **14**(2): p. 345-355.
9. Kim, T., et al., *Computational analysis of viscoelastic properties of crosslinked actin networks.* PLoS computational biology, 2009. **5**(7): p. e1000439.
10. Veigel, C., et al., *Load-dependent kinetics of force production by smooth muscle myosin measured with optical tweezers.* Nat Cell Biol, 2003. **5**(11): p. 980-6.
11. Uemura, S., et al., *Mechanochemical coupling of two substeps in a single myosin V motor.* Nat Struct Mol Biol, 2004. **11**(9): p. 877-883.
12. Jung, W., M.P. Murrell, and T. Kim, *F-actin cross-linking enhances the stability of force generation in disordered actomyosin networks.* Comp Part Mech: p. online first.
13. Kovacs, M., et al., *Mechanism of blebbistatin inhibition of myosin II.* J Biol Chem, 2004. **279**(34): p. 35557-63.
14. Limouze, J., et al., *Specificity of blebbistatin, an inhibitor of myosin II.* J Muscle Res Cell Motil, 2004. **25**(4-5): p. 337-41.
15. Marchetti, M.C., et al., *Hydrodynamics of soft active matter.* Reviews of Modern Physics, 2013. **85**(3).
16. Banerjee, S. and M.C. Marchetti, *Instabilities and oscillations in isotropic active gels.* Soft Matter, 2011. **7**(2): p. 463-473.
